# Telomere Maintenance Mechanisms in Cancer

**DOI:** 10.3390/genes9050241

**Published:** 2018-05-03

**Authors:** Tiago Bordeira Gaspar, Ana Sá, José Manuel Lopes, Manuel Sobrinho-Simões, Paula Soares, João Vinagre

**Affiliations:** 1 Cancer Signaling and Metabolism Group, Institute for Research and Innovation in Health Sciences (i3S), University of Porto, 4200-135 Porto, Portugal; tgaspar@ipatimup.pt (T.B.G.); asa@ipatimup.pt (A.S.); jmlopes@ipatimup.pt (J.M.L.); ssimoes@ipatimup.pt (M.S.-S.); jvinagre@ipatimup.pt (J.V.); 2 Cancer Signaling and Metabolism Group, Institute of Molecular Pathology and Immunology of the University of Porto (Ipatimup), 4200-135 Porto, Portugal; 3 Medical Faculty of University of Porto (FMUP), 4200-139 Porto, Portugal; 4 Abel Salazar Biomedical Sciences Institute (ICBAS), University of Porto, 4050-313 Porto, Portugal; 5 Department of Pathology and Oncology, Centro Hospitalar São João, 4200-139 Porto, Portugal

**Keywords:** cancer, telomere, telomerase, promoter, *TERT*, *TERC*, alternative lengthening of telomeres (ALT), telomere maintenance mechanism (TMM), non-defined telomere maintenance mechanism (NDTMM)

## Abstract

Tumour cells can adopt telomere maintenance mechanisms (TMMs) to avoid telomere shortening, an inevitable process due to successive cell divisions. In most tumour cells, telomere length (TL) is maintained by reactivation of telomerase, while a small part acquires immortality through the telomerase-independent alternative lengthening of telomeres (ALT) mechanism. In the last years, a great amount of data was generated, and different TMMs were reported and explained in detail, benefiting from genome-scale studies of major importance. In this review, we address seven different TMMs in tumour cells: mutations of the *TERT* promoter (*TERTp*), amplification of the genes *TERT* and *TERC*, polymorphic variants of the *TERT* gene and of its promoter, rearrangements of the *TERT* gene, epigenetic changes, ALT, and non-defined TMM (NDTMM). We gathered information from over fifty thousand patients reported in 288 papers in the last years. This wide data collection enabled us to portray, by organ/system and histotypes, the prevalence of *TERTp* mutations, *TERT* and *TERC* amplifications, and ALT in human tumours. Based on this information, we discuss the putative future clinical impact of the aforementioned mechanisms on the malignant transformation process in different setups, and provide insights for screening, prognosis, and patient management stratification.

## 1. Introduction

Gradual accumulation of genetic errors in cells is a major contributor to the tumourigenic process. In the transition to a malignant tumour (i.e., cancer), an acquired immortality state is mandatory, and tumour cells must cope with selective pressure. It is therefore required that cancer cells gain advantages against tumour suppressive mechanisms. Limiting telomere shortening is one of those mechanisms, being the topic of this review.

Telomeres are DNA–protein complexes at the ends of eukaryotic chromosomes that play a crucial role in cellular survival, by limiting progressive loss of genomic information caused by semi-conservative replication of DNA [1,2]. Most cancer cells maintain the integrity of their telomeres by telomerase reactivation (TR) [3], and the mechanisms accounting for telomere length (TL) maintenance are currently known to comprise transcriptional, post-transcriptional, and epigenetic regulation [4,5]. A small part of tumour cells acquires immortality through the alternative lengthening of telomeres (ALT) mechanism. An understanding of these mechanisms and respective age- and tumour-related changes will hopefully unveil novel biomarkers and targets with diagnostic and prognostic impact, and ultimately influence the development of novel therapeutics [3,6].

In this review, we address seven telomere maintenance mechanisms (TMMs) in tumour cells, including genetic (promoter mutations, amplifications, germline genetic variations, rearrangements) and epigenetic (DNA methylation and non-coding RNAs) events.

## 2. Telomere Maintenance Mechanisms 

### 2.1. Telomere Maintenance Mechanisms in Non-Malignant Cells

Telomeres are specialized ribonucleoprotein structures composed of DNA and bound proteins localized at the ends of all linear chromosomes [7,8]. Telomeric DNA contains a multiple short non-coding tandem repeat of double-stranded DNA sequence, 5′-(TTAGGG)_n_-3′ that is 10–15 kilobases (kb) long in humans at birth, and a 3′ G-rich single-stranded tail of 150–200 nucleotides [9,10].

The proteins associated with telomeres comprise the shelterin complex that promotes telomere protection, by ensuring stability and assisting specialized replication machinery for accurate extension of chromosome ends [7,10,11] and recruitment of telomerase [8,12,13]. The shelterin complex consists of six subunits, three DNA-binding (TRF1, TRF2, POT1) interconnected by three additional proteins (TIN2, TPP1, RAP1) that act as adaptors and mediate interactions among the constituents [14,15].

Telomeres play vital roles in dealing with two unavoidable biological challenges, the end protection—by safeguarding chromosomes from being recognized as double stranded free DNA breaks by the DNA damage response (DDR) machinery, that may result in end-fusions and genome instability [12]—and the end replication crises—the inherent limitation of DNA replication, i.e., the gradual shortening of DNA at chromosomal ends at each replicative cycle [8,10].

Telomerase is a complex ribonucleic reverse transcriptase responsible for synthetizing telomeric DNA repeats at the 3′ ends of linear chromosomes [9,15,16]. It comprises the catalytic protein subunit telomerase reverse transcriptase (TERT), encoded by the *TERT* gene (located at 5p15.33), an essential RNA component (*TERC*) that functions as the RNA template for the addition of telomeric repeats, encoded by the *TERC* gene (at chromosome 3q26) [3,4,17], and a series of auxiliary components with important biologic functions that include dyskerin, reptin, pontin, and ribonucleoproteins NOP10, GAR1, and NHP2 [15,18,19]. TERC, additionally to its role in the template for the synthesis of telomere DNA, is also involved in the catalysis, localization, and assembly of telomerase [20].

Defects in these telomerase players are known to cause telomere deficiency syndromes or telomeropathies, as reviewed by some authors [9,21,22].

Telomere length in stem cells is established with a relative size that grants tissue homeostasis during embryogenesis but is short-limited enough to suppress unlimited cell proliferation and tumour initiation [23]. As proliferating cells of self-renewing tissues depend on telomerase activity as a pivotal TMM, most human somatic tissues do not express sufficient telomerase to infinitely sustain TL, leading to gradual telomere shortening [24,25]. Therefore, cells undergo gradual age-related telomere shortening, at a variable rate per mitosis [9,26]. Gradual telomere attrition reflects one of the hallmarks of aging [27].

As reviewed by Jafri et al. [4], telomerase is responsible for a multistep process required for telomere maintenance, that includes TERT protein transport and trafficking into the nucleus, TERC and TERT assembly with accessory components in the nucleus, and recruitment to the telomeres at the correct timing during DNA replication. Repressors and enhancers within *TERT* promoter engage in a transcriptional suppression of the catalytic subunit in most somatic cells, thus limiting telomerase activity [15,28].

Telomere length is also regulated by epigenetic marking in telomeric chromatin [29,30]. The compacted chromatin state of mammals, which contains histone modifications suggestive of a constitutive heterochromatin, negatively regulates TL [31]. When these heterochromatic marks are lost, telomere elongation occurs, as reported in mouse cells, suggesting that a compacted chromatin state at telomeres is fundamental for controlling TL; i.e., the compaction of chromatin and subsequent difficult access of transcription factors may induce negative regulation of TMMs [30,31,32].

As telomeres shorten, they can also modify at a transcriptionally level the expression of nearby genes, telomere position effect (TPE), or over long distant genes (TPE-OLD) [33]. Telomere position effect involves the spreading of telomeric heterochromatin to silence genes in the vicinity of telomeres according to TL, while TPE-OLD telomeres fold back and physically interact with other chromosome domains, producing widespread changes in gene regulation much sooner than TL decreases above a critical level to induce DDR [33].

Independent of the reactivation of telomerase, ALT represents a TMM based on homologous recombination (HR) and homology-directed telomere synthesis [34,35] that was thought to be exclusive of tumour cells; still, it has been identified in stem cells and healthy tissues of mouse [31,36]. It has also been detected in human cells of the placenta in early gestation [37] and endothelial, stromal, and some epithelial cells of non-neoplastic cells [38]. This mechanism might thus occur naturally in another physiological setting that is not fully understood at this point and can be a recombination-based mechanism. Finally, telomere sequences contain long non-coding RNAs—telomeric repeat-containing RNA (TERRA)—with important functions on telomere homeostasis and telomerase function [39], that will be further addressed.

### 2.2. Telomere Maintenance Mechanisms in Tumour Cells

The ability to keep telomeres above a critical length represents a vital feature of malignant cells [40]. Activation of a TMM, dependent or independent of the enzyme telomerase, allows tumour cells to survive cellular crisis and achieve immortality, one of the major hallmarks of cancer [41,42,43].

Both *TERT* and *TERC* codify limiting protein components of telomerase activity [44]. Transcription, alternative messenger RNA (mRNA) splicing, phosphorylation, maturation, and modification of TERT and of TERC have been reported to play vital roles in the regulation of telomerase activity [3]. 

Concurring with tumour heterogeneity, TL is also expected to fluctuate [45]. It was reported that genes closer to telomeres display higher expression in tumours than in normal tissues, due to the reduced TL of the first; and this effect seems gradually attenuated as distance to telomeres increases [45]. 

The central role of TMMs in cancer led to the development of several therapeutic strategies aiming at inhibiting telomerase and/or telomere function, such as the use of small-molecule telomerase inhibitors, oligonucleotide inhibitors, immunotherapy, and G-quadruplex stabilizers [46,47,48]. 

Telomere maintenance in tumour cells is ensured by TR in over 85% [45,49,50,51,52,53,54] of human tumours, while ALT mechanism occurs in 10–15% [35,55,56].

**Figure 1:**
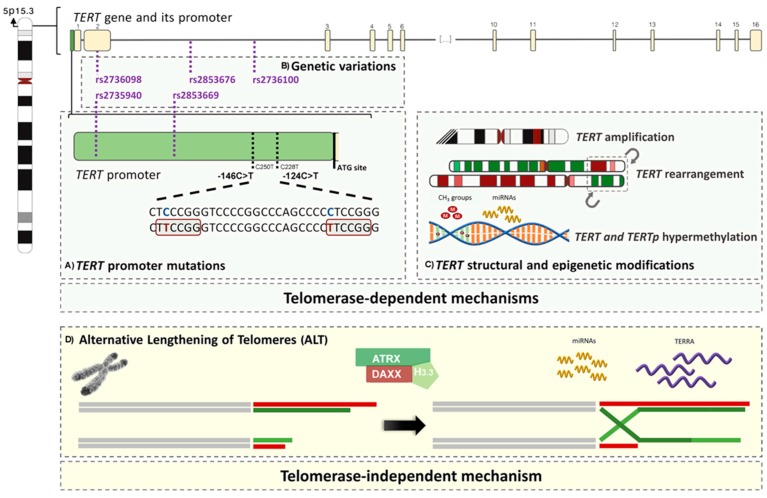
Telomerase-dependent (**A**,**B**) and -independent (**C**) telomere maintenance mechanisms (TMM) in cancer. Telomerase reactivation depends on several mechanisms that imply modifications that can have a direct impact on *TERT* gene regulation, which is localized at the short arm of chromosome 5. (**A**) *TERTp* hotspot mutations (−124 bp and −146 bp upstream the ATG transcriptional start site) create binding sites for ETS transcription factors (red boxes); (**B**) Germline genetic variations of the *TERTp* and of intronic and exonic regions seem to associate with cancer risk; their genomic coordinates based on build 37 (GRCh 37, hg19/Human); (**C**) *TERT* structural variants comprise amplification and rearrangement of the gene. Hypermethylation of the *TERTp* or other regions, micro RNA (miRNA) regulation and post-translational histone modifications are epigenetic modifications involved in telomerase reactivation; (**D**) Alternative lengthening of telomeres (ALT) is a telomerase-independent mechanism that relies on the homologous recombination machinery of DNA repair to maintain telomere length. Mutation of the genes *ATRX* or *DAXX* and loss of protein expression are known events related to ALT. miRNAs and TERRA molecules are some epigenetic regulators of ALT. *TERT*: telomerase reverse transcriptase; *TERTp*: *TERT* promoter.

The most characterized mechanisms and alterations (Figure 1) responsible for maintaining the lengthening of telomeres in tumour cells are: (1) somatic mutations of the *TERT* promoter (*TERTp*); (2) amplification of the genes *TERT* and *TERC* (3) rearrangements of the *TERT* gene; (4) germline genetic variants of the *TERT* gene and its promoter; (5) epigenetic changes; (6) ALT; and (7) non-defined TMM (NDTMM).

The recent study by Barthel et al. [45] highlighted the telomere length and frequencies of telomere maintenance by mechanism and tumour type in The Cancer Genome Atlas (TCGA) cohort. By analysing the data from 288 papers, we collected the percentages of occurrence of five different TMMs (*TERTp* mutations, *TERT* and *TERC* amplifications, *TERT* rearrangements, and ALT) from over fifty thousand cases.

The different TMMs are extremely diverse amongst several tumours in different locations and histotypes. When considering large cohorts (more than 100 patients) the tumours with the highest prevalence of *TERTp* mutations are glioblastoma (GB) *IDH*-wildtype (72%), oligodendroglioma (OD) *IDH*-mutant and 1p19q-codeleted (95%), anaplastic oligodendroglioma (AOD) (63%), adult sonic hedgehog medulloblastoma (SHH-MB, 89%), hepatocellular carcinoma (HCC, 41%), oral squamous cell carcinoma (SCC) (50%), basal cell carcinoma (BCC) and SCC of the skin (49% and 56% respectively), metastatic cutaneous melanoma (76%), urothelial bladder carcinoma, both non-muscle invasive (NMIBC, 69%), and muscle invasive (MIBC, 68%), that are in contrast to tumours with high cell turnover that present less prevalence of *TERTp* mutations, e.g., tumours of the digestive system (0–2%) and haematopoietic and lymphoid tissues (0%). 

Adenocarcinoma and SCC of the lung (18% and 40%, respectively) contrast with oral SCC (2%) concerning the presence of *TERT* amplifications in cohorts with more than 200 patients. Cervical intraepithelial neoplasia (CIN) presents a prevalence of *TERC* amplifications that increases with progression: 24%, 69% and 88% (CIN1, 2, and 3, respectively). Lung SCC was also reported with a high frequency of *TERC* amplifications (41%).

High-risk and high-stage neuroblastoma (NBL) (15%) is, so far, the best characterized tumour model for rearrangements of the *TERT* gene (cohort of 292 patients). 

ALT mechanism also exhibits lower rates in tumours of the digestive and haematopoietic systems, while neuroblastomas (50%) and osteosarcomas (63%) frequently display this phenotype (cohorts with more than 100 patients). A large cohort of patients with pancreatic neuroendocrine tumours (pNETs) were reported to display 30% of ALT positivity. Sporadic pNETs often present ALT, whereas *TERTp* mutations are detected in a fraction of hereditary pNETs [57]. 

These data portray the diverse panoply by which TMMs can be found in human tumours. For the sake of simplicity, we will address each tumour histotype, whenever available, according to the current World Health Organization (WHO) classification, for the several organ/tissue locations. When discrete histotypes were not available or the reported cohorts included few cases, we included them in a not otherwise specified (NOS) group, indicating tumour histotypes as reported by the authors (Table 1, Table 2, Table 3 and Table 4). Molecular associations, prognostic, and clinical implications of TMMs in human tumours are summarized in Table 5. Information regarding the distribution of absent/low frequency TMMs in prevalent tumours can be consulted in Table 6. 

#### 2.2.1. *TERT* Promoter Mutations

Somatic mutations in the coding region of *TERT* are not frequent in tumours. Somatic mutations in the *TERTp*, however, have recently emerged as the most prevalent non-coding mutations in human cancer.

*TERTp* is a 260 bp region that lacks a TATA box or other similar sequence, containing binding motifs for various transcription factors that regulate gene transcription and are responsible for *TERT*’s transcriptional activity and telomerase activation [3,4].

Independent somatic mutations in the core promoter of *TERT* were recently reported in melanoma [189,190]. The most frequent mutations resulted in a C>T transition at −124 bp and −146 bp upstream the ATG transcriptional start site; these hotspot mutations are also known as C228T and C250T, respectively, based on their genomic coordinates. Both create an 11 bp nucleotide stretch that contains a consensus binding site for E-twenty-six (ETS) transcription factors within the promoter region. This provides a basis for the biological relevance of these mutations, since ETS transcription factors may be activated through dysregulation of mitogen-activated protein kinase (MAP kinase) signalling, commonly observed with increased gene expression in some tumours [189,190,343]. Tumours with *TERTp* mutations were consistently reported to express higher levels of TERT mRNA and telomerase activity when compared to those with a wildtype promoter [51,75,189,190,204,344,345]. 

The level and frequency of these mutations varies greatly between tumour histotypes [3]. After their first report in melanoma, *TERTp* mutations have been reported in many other types of tumours, such as tumours of the central nervous system (CNS), thyroid, skin, and liver [49,51,70,96,116,172,202,344,346]. This neoplastic spectrum led to the postulation that *TERTp* mutations preferentially trigger tumorigenesis in tissues with relatively low rates of self-renewal, that are not able to overcome the short-telomere dependent proliferative barrier, and arise as a late tumorigenic event [4,49]. Still, *TERTp* mutations can also appear as an early tumorigenic event, resulting from environmental factors, namely, ultraviolet radiation or chemical carcinogens. This possibility is supported by their high prevalence in BCC, melanoma, and urothelial bladder carcinoma [22]. 

The frequencies of *TERTp* mutations were reviewed by tumour histotype as depicted in Table 1. Regarding diffuse astrocytic and oligodendroglial tumours, gliosarcomas present the highest mutation rate (81%), followed by glioblastomas *IDH*-wildtype (72%). The prevalence of *TERTp* mutations varies considerably between primary/secondary (68% vs. 25%) and adult NOS/paediatric GBs (63% vs. 6%). *IDH*-mutant and 1p19q-codeleted anaplastic oligodendrogliomas present a lower prevalence *TERTp* mutations (21%) when comparing to NOS histotypes (63%). Anaplastic oligoastrocytomas (AOA, WHO grade III) also have a high mutational rate (41%). Pilocytic astrocytomas (WHO grade I) exhibit low or absent *TERTp* mutations. Within embryonal tumours, *TERTp* mutations occur in SHH adult medulloblastomas (89%) and in paediatric SHH-MB (34%). These incidences suggest that *TERTp* mutations correlate with patient age, as consistently described in studies of CNS tumours, mainly GB [49,50,66,74,98,347]. *TERTp* mutations seem to be consistently associated with WHO high-grade in CNS tumours. Additionally, it seems that *TERTp* mutations influence the clinical outcome, since they have been reported to be linked with tumour progression and to predict a worse survival in patients with CNS tumours [65,80,98,346,348].

The association of *TERTp* mutations with other genetic alterations in CNS tumours needs to be clarified. Isocitrate dehydrogenase (*IDH*) mutations are common in diffuse astrocytic and oligodendroglial tumours. The combined analysis of different histotypes regarding *IDH* and *TERTp* mutational status led to the conclusion that *TERTp* mutations are more prevalent in *IDH*-wildtype tumours, such as in GBs (72% in *IDH*-wildtype vs. 24% in *IDH*-mutated tumours) and anaplastic astrocytomas (AA) (47% vs. 11%); diffuse astrocytomas present a smaller variation (26% vs. 16%). Remarkably, in ODs *IDH*-mutated and 1p19q-codeleted, *TERTp* mutations are associated in a much higher extent to WHO grade II, OD (95%) than in WHO grade III, AOD (21%). 

Throughout recent years, several authors have proposed the combination of *TERTp* mutations with other CNS tumour-associated molecular features, namely *IDH* mutations, 1p19q loss, and O6-methylguanine-DNA-methyltransferase (O6-MGMT) methylation status, willing to establish a molecular signature that could assist more efficiently in defining an accurate prognosis and the best therapeutic option in patients with diffuse astrocytic and oligodendroglial tumours. The combination of *TERTp* and *IDH* mutations, in particular, allows the assignment of diffuse astrocytic and oligodendroglial tumours in discrete groups with different survival rates: *TERTp* and *IDH*-mutated tumours present the longest overall survival, while patients with only *TERTp*-mutated tumours present the lowest survival, as shown in several reports [61,71,73,74,78,79]. Triple positive ODs (*TERTp*-, *IDH*-mutated, and 1p19q-codeleted) are associated with better overall survival [71].

In tumours of the digestive system, the highest mutation rates occur in hepatocellular carcinoma (41%) and gallbladder carcinoma (9%). *TERTp* mutations are absent in hepatocellular adenoma (HCA, 0%); noteworthy, borderline HCA/HCC and HCC derived from HCA present high mutational rates, 48% and 17%, respectively, which suggests the potential involvement of *TERTp* mutations in the malignant transformation of HCAs [95,96,349]. 

In some studies, *TERTp* mutations represent the most common genetic event in HCC [49,96], but other reports present a lower prevalence, which can be due to the studied populations since the mutation frequency varies according to the geographic region [350] and is linked with the prevalence of hepatitis B or C viral infection. *TERTp* mutations were reported in HCC associated with both viruses, with a higher prevalence for the hepatitis C virus [99,106,351]. Other evaluated tumours of the digestive system presented extremely low or absent prevalence of *TERTp* mutations, e.g., gastric carcinoma, tumours of the colon and rectum and exocrine pancreas, pointing to a minor role of *TERTp* mutations in these tumours.

*TERTp* mutations are highly prevalent in thyroid tumours. The highest mutational rate occurs in aggressive thyroid carcinoma histotypes: anaplastic thyroid carcinoma (ATC, 46%) and poorly differentiated thyroid carcinoma (PDTC, 41%), followed by metastases of well-differentiated papillary and follicular carcinomas (21%), follicular thyroid carcinoma (FTC, 18%), and papillary thyroid carcinoma, NOS (PTC, 11%). Specific subtypes of PTC, such as tall cell variant (TCVPTC), show a higher frequency (19%), consistent with the more aggressive behaviour of this variant of PTC. PTC with *TERTp* mutations consistently associate with larger tumours, older patient age, higher tumour stage, tumour recurrence, and distant metastases [51,116,119,125,138,333]. *TERTp* mutations associate with *BRAFV600E* mutations [117,126,141,146,352]. *TERTp* mutations are not detected in normal thyroid tissue and the studies in benign tissues and benign thyroid tumours (3%) point to a low frequency; however, the prevalence rises in settings of atypical follicular adenomas (17%) [115] and X-ray irradiation (21%) [139].

Papillary carcinoma-derived anaplastic carcinomas were recently characterized by the co-occurrence of *BRAFV600E* and *TERTp* mutations prior to anaplastic transformation. This led the authors to suggest that PTC harbouring *TERTp* mutations have higher risk of anaplastic transformation [142]. Paediatric thyroid tumours do not seem to harbour these mutations [150,151], which parallels the findings regarding specific subtypes of CNS tumours. At variance with follicular cell derived tumours of the thyroid, medullary thyroid carcinomas do not harbour *TERTp* mutations. 

Within the female reproductive organs, *TERTp* mutations occur in clear cell carcinoma (CCC) of the uterus and the ovary (21% and 16%, respectively). In ovarian CCC, the mutations were correlated with patient age [153], but not with disease-specific survival [152]. The mutational frequency for uterine endometrial carcinoma, low-grade serous carcinoma of the ovary, and cervical SCC is 11%, 5% and 15%, respectively. 

Concerning haematopoietic and lymphoid tissues tumours, *TERTp* mutations were, so far, detected only in a study of mantle cell lymphomas (22%) [161]. 

With regard to skin tumours, BCC harbours 49% of *TERTp* mutations with a UV-signature, indicating UV exposure as a potential cause in these frequent tumours [170,172], being the same signature shared by SCC that harbours 56% *TERTp* mutations [154]. In cutaneous melanomas, *TERTp* mutations were reported in several histotypes: superficial spreading (34%), nodular (55%), lentigo maligna (24%), acral lentiginous (9%), and desmoplastic (45%). Metastatic cutaneous melanoma presents a remarkable rate of mutations (76%), as well as metastatic melanoma of unknown primary sites (49%) and of other primary locations (54%). In cutaneous melanomas, the mutations associate with male gender [184,185], older age, tumours with ulceration, and higher Breslow’s thickness [92,175,179,184,221]. In some studies [182,184], the *TERTp* mutations were not associated with clinical outcome, at variance with the results of Griewank et al. [92] and Pópulo et al. [171], who reported an association between *TERTp* mutations and poorer overall survival in patients with cutaneous melanomas. The occurrence of *TERTp* mutations in cutaneous melanoma associates with *BRAF* mutations and with poorer prognostic features, such as higher Breslow’s thickness, higher mitotic rate, presence of ulceration, absence of regression, and lymph node metastases [92,171,174,190,221]. The *BRAF* and *TERTp* mutations may cooperate in cutaneous melanoma and recent evidence indicates that their combination can be used to identify tumours with aggressive behaviour [175,179,186]. Atypical fibroxanthoma and pleomorphic dermal sarcoma are genetically poorly understood and were reported to exhibit a high frequency of *TERTp* mutations (93% and 77%, respectively), which stands as the most frequent genetic event in such tumours [191]. No mutations were reported either in cutaneous or conjunctival naevi, consistent with a putative late pathogenic role of *TERTp* mutations in the progression of these melanocytic tumours [90]. 

*TERTp* mutations are highly frequent in urinary system tumours. They are the most frequent alterations in both invasive (68%) and non-muscle invasive (69%) urothelial cell carcinoma of the bladder. Their presence is of limited prognostic value due to the equal mutational rate in different stages or grades [208] but may be a useful biomarker for urine-based tumour monitoring (non-invasive diagnostic tool in two settings: early detection in high-risk patients and recurrence in patients with urothelial bladder carcinoma [203,206,209]. High telomerase activity has been correlated with urothelial carcinomas as a better marker of disease aggressiveness than *TERTp* mutations alone [344], but when combined with *FGFR3* mutations, these mutations assist on establishing tumours of poor prognosis [222]. In kidney tumours, *TERTp* mutations are less prevalent, with a frequency of 9% in clear cell renal cell carcinoma (ccRCC, 9%). The mutations in ccRCC are less prevalent than in many other tumour types, but their presence was reported to characterize a subset of tumours that demand more aggressive treatment [198]. *TERTp* mutations were not reported, so far, in prostate cancer [49,168,201,219,353].

Chiba et al. [354] recently proposed the contribution of *TERTp* mutations to tumorigenesis in a two-step mechanism. The authors reported that in an initial phase, *TERTp* mutations heal the shortest telomeres and extend life-span; in a second phase, genome instability arises as a consequence of critically short telomeres, inducing the upregulation of telomerase to sustain cell proliferation [354]. The selection of *TERTp* mutations at the transition from pre-neoplastic to malignancy suggests that telomere shortening acts as a critical barrier early in the tumorigenesis of some cancers. 

*TERTp* mutations currently stand as highly prevalent events in a spectrum of tumours, with varying rates according to histotype (Table 1). They present a relevant role in hepatocarcinogenesis and also stand as a powerful tool with impact on clinical management, namely screening of patients with urothelial carcinoma of the bladder, or prognosis stratification of patients with diffuse astrocytic and oligodendroglial, thyroid, and skin tumours.

#### 2.2.2. *TERT* and *TERC* Amplifications

Copy number variations (CNVs), or alterations (CNAs), affect a larger fraction of the genome in cancers than do any other type of somatic genetic alteration [355,356,357,358]. CNVs are more successfully detected by next generation sequencing [45], rather than by fluorescence in situ hybridization (FISH) studies used in the past [359].

Gain or loss of genetic material is commonly observed in human malignancies [17,52,360]. DNA amplification is a frequent event in the tumorigenic process and typically culminates in gene overexpression [17,52,235,238,361]. 

Some authors reported CNVs of whole chromosomes or chromosome arms in a large number of human tumours. In the year 1999, Rooney et al. [360] identified the chromosome arms 3q (16.4%) and 5p (13.2%), respectively, as the sixth and eighth regions where more frequently chromosomal gains occurs, by comparative genomic hybridization (CGH) in 2210 solid tumours of 27 tumour types. As reviewed by Soder et al. [238], Knuutila et al. [362], and Sugita et al. [235], amplifications in 3q have been associated to many tumour types, including ovarian carcinoma [363,364,365], cervical carcinoma [366,367], lung carcinoma (both small cell [368,369,370,371] and non-small cell [365,372]), SCC of the head and neck (SCC-HN) [372,373,374], and non-Hodgkin lymphoma [375]. Amplifications in 5p have also been detected in cervical carcinoma [367], non-small cell lung cancer (NSCLC) [376], SCC-HN [374], and in many other tumours, as osteosarcoma [377], malignant fibrous histiocytoma of soft tissue [378], and gastrointestinal stromal tumour (GIST) [379]. 

Considering that *TERT* has been mapped to chromosome 5 at 5p15.33 and *TERC* has been mapped to chromosome 3 at 3q26.3 [238,359], authors started to search for specific changes in copy number. Both amplifications were reported to be genetic alterations that induce strong TERT [52,163,229,380] and TERC [238,359] overexpression in some tumours, although the specificity of these amplifications remains to be established [238]. Noteworthy, HCC [226] and malignant pleural mesothelioma [163] are tumours in which *TERT* gene is overexpressed but not 9ssociated with gene copy number gain. 

Increased *TERT* and *TERC* gene dosage has been detected frequently in a variety of human tumours, and clonal evolution of cells with increased *TERT* or *TERC* copy number has been observed, pointing towards a growth advantage in cells with increased *TERT* or *TERC* gene dosage [17]. Below, we present the prevalence of *TERT* and *TERC* amplifications in several tumour types. For histotype information, report to Table 2. 

In cutaneous melanomas (Table 2), an increase of CNVs comes with tumour progression, as *TERT* amplifications were detected only in invasive melanomas, whereas they were rarely detected in benign naevi, and occasionally present in intermediate lesions, melanomas in situ, and desmoplastic melanomas. *TERT* amplifications were reported in 23% of acral-lentiginous melanomas and less than 5% in desmoplastic melanomas.

Despite the already stated role of *TERTp* mutations in hepatocarcinogenesis as an early event in tumour progression and the cause of higher TERT expression [99,103], *TERT* amplification does not show a clear correlation with progression. In HCC, *TERTp* mutations and *TERT* focal amplifications are almost mutually exclusive [99]. Overall, amplifications of the *TERT* gene were detected in 15% of HCC.

In addition to HCC, *TERT* amplifications presented a substantial prevalence in lung adenocarcinoma (18%) and lung SCC (25%), colorectal carcinoma (48%), and cervical intraepithelial neoplasm (CIN) 2 and 3 (60% and 88%, respectively).

Besides desmoplastic cutaneous melanomas, *TERT* amplifications presented the lowest levels in phyllodes tumour of the breast (4%) and oral SCC (2%).

*TERT* amplifications have also important utility in diagnosis of a variety of solid tumours, including breast (differentiating phyllodes tumours from fibroadenomas) [223], NSCLC [381] and urothelial bladder carcinomas [234]. Also, *TERT* amplifications may represent a poor prognostic marker in breast [382] and urothelial bladder carcinomas [234], NSCLC [229], and acral-lentiginous melanoma [231].

Amplifications of the *TERC* gene (Table 2) were reported at high levels in 21% of oesophageal carcinoma [45], 41% of lung SCC [45], ovarian (37%), and cervical tumours (59%) [45,235]. Andersson et al. [240] wrote the first report of consistent gain of *TERC* in cervical adenocarcinoma. In what concerns CINs, both *TERC* and *TERT* amplifications can be accurately detected with FISH technique in routinely collected liquid based cytology (LBC) by Pap smears [227,236,237].

Gains of *TERC* gene significantly associate with a gradual increasing amplification pattern in tumour progression of ovarian malignancies [227,236,237,383,384]. Visnovsky et al. [227] reported for *TERT* gene a very similar amplification pattern that also associates with histopathological and cytopathological findings. Liu et al. [236] reported a significant positive correlation between the level of *TERC* amplification and histologic grade of intraepithelial cervical lesions: lower in low-grade (CIN 1) than in high-grade (CIN 2/3). Both authors describe *TERC* amplification in all cases of malignant cervical carcinomas evaluated. 

Concerning urothelial bladder carcinomas, amplification of the *TERT* gene appear to be useful in discriminating patients with non-muscle (0%) and muscle invasive (56%) tumours in the study of Yamamoto et al. [234].

In conclusion, *TERC* and *TERT* amplification assessment may be useful in the future as a complement to the HPV test and help to establish the risk of malignancy of cervical precursor lesions, aiming the highest combined sensitivity and specificity [227,236,237], i.e., early identification of patients with low-grade lesions and higher progression risk [384]. 

Copy number variations represent an additional mechanism for telomere maintenance based on the capacity to modulate telomerase overexpression and for some tumour types it was reported a mutual exclusivity towards other TMMs. 

#### 2.2.3. *TERT* Germline Genetic Variations

The 5p15.33 *TERT*-*CLPTM1L* chromosomal region has been consistently associated with susceptibility for multiple tumours [385]. Genome-wide association studies (GWAS) performed in large scale in the last decade have strongly contributed to the identification of common variants in *TERT* locus. Several single nucleotide polymorphisms (SNPs) in this region have been consistently associated with increased risk for developing various types of tumours. These SNPs may arise either in intronic or exonic sequences of *TERT* or in its promoter. The more described genetic variants for both regions of *TERT* and their associations with several types of cancers are depicted in Table 3; most studies are genome wide associations where the results are not available by histotypes. 

The polymorphism rs2736100 is localized in intron 2 of *TERT* and it is one of the most described variants of the gene. It has been thoroughly associated with lung cancer risk (mostly adenocarcinoma [242,249]), although there were also reports of no evident association [386]. Wang et al. [387] and Yang et al. [388] published meta-analyses in which they reported the association of rs2736100 with increased lung cancer risk (mainly adenocarcinoma) in both Caucasian and Asian populations. This SNP was identified to associate with an increased risk of myeloproliferative neoplasms (e.g., polycythaemia vera, essential thrombocythemia, and primary myelofibrosis) in Caucasian and Chinese populations [267,269]. Conflicting results have been reported regarding the effect of this SNP on gastric cancer risk, in which rs2736100 was associated with increased risk in a Turkish population [265] but did not show impact on an Asian population, in which it correlated only with the regulation of TERT expression and telomere length [389]. On the other hand, rs2736100 was significantly associated with reduced risk of upper tract urothelial carcinoma [390] and increased prostate cancer aggressiveness [391]. No association was reported for this SNP and colorectal cancer [274] or breast cancer risk [276]. Given the conflicting and heterogeneous results obtained from various studies, several meta-analyses have been published to address the effects of the polymorphism on cancer risk. Zou et al. [392] reported a significant association between rs2736100 and cancer susceptibility, with strong associations for lung and pancreatic cancers and BCC, and also risk alleles for bladder, prostate and cervical cancers, as well as gliomas (including WHO grades II–IV astrocytic, and WHO grades II–III oligodendroglial tumours). Li et al. [393] reported that rs2736100 polymorphism in heterozygous and homozygous variant genetic settings affected cancer susceptibility from a gathering of 16 case-control studies. The evaluated studies reported discrepancies that could be explained by different allele frequencies in different ethnicities. Peng et al. [335] evaluated the association between rs2736100 and the risk of glioma (including WHO grades II–IV astrocytic and WHO grades II–III oligodendroglial tumours) from 16 independent studies and reported that this genetic variation may greatly enhance susceptibility for developing these types of tumours, with consistent results obtained for Caucasians. The authors emphasized the need of more studies for Asian populations and pointed to the fact that the heterogeneity found could be attributed to genetic backgrounds, living environments and patients’ characteristics. 

The rs2736098 is a synonymous coding SNP in the second exon of the *TERT* gene that was associated with telomere length but not TERT expression [271]. Rafnar et al. [271] reported its association with increased risk of BCC and lung, prostate and bladder cancers. This variant has also been reported not to associate with breast cancer risk [288] and to reduce the risk of SCC-HN and oral cavity in Caucasian patients [266,394]. Wu et al. [395] demonstrated that this polymorphism may contribute to the risk of lung cancer (especially adenocarcinoma), but they found it to be only weakly associated with overall cancer risk.

The rs2853676 maps to intron two of *TERT* [243]. Other types of cancer than the ones detailed in Table 3 (breast, gastric, lung, prostate and ovary; gliomas; and melanomas) have been investigated regarding this SNP, and no associations were found for endometrial cancer [396], BCC or SCC of the skin [279], colorectal cancer [397,398], breast cancer [399] or paediatric acute lymphoblastic leukaemia [256]. Cao et al. [336] performed a systematic review and meta-analysis to ascertain the impact of rs2853676 on cancer risk, and they reported association with increased risk of glioma (including WHO grades II-IV astrocytic and grades II-III oligodendroglial tumours), lung and ovarian cancers among Caucasian populations.

The rs2853669 functional variant is located in the *TERTp* within a binding site for ETS2 transcription factor [385]. It was reported to affect telomerase activity and telomere length [275,400]. Rs2853669 appears to be associated with prognosis, affecting survival and tumour recurrence in urothelial bladder carcinoma, although these results are conflicting among different studies [204,222]. Depending on the rs2853669 SNP status, glioblastoma patients carrying *TERTp* mutations displayed worse prognosis and shorter survival [68,76,80]. This polymorphism has also been consistently associated with cutaneous melanoma patients carrying *TERTp* mutations [188,293], and its use to identify patients at risk of aggressive disease was proposed [175]. Beyond association with increased cancer risk and cancer prognosis *per se*, rs2853669 has been reported to have a modifying effect on *TERTp* mutations [65,76,80,175,198,204,292,401], and an eventual prognostic value [402].

TERTp polymorphism rs2735940 acts similarly but is reported in less extent than the rs2853669. The association with cancer risk has been found for lung and gastric cancers [265,294], as well as for paediatric acute lymphoblastic leukaemia [256]. No association between this polymorphism and colorectal cancer or colonic polyps has been found [397], although these results are conflicting among studies [403].

*TERT* polymorphisms are being addressed as factors with impact on the risk of developing several cancers, with increasing evidence for tumours of the CNS and lung. However, there are still conflicting reports, which renders this a debatable issue. It should be kept in mind that a different genetic background and/or racial and ethnic disparities may play important roles in the pathogenesis and modulate disease incidence. *TERTp* polymorphisms, their reported modifying effect on *TERTp* mutations, and their use in patient prognosis are also an interesting target to be further explored.

#### 2.2.4. *TERT* Rearrangements

Chromosomal rearrangements are another TMM that occur in human tumours. Genomic rearrangements can result in tandem duplications, inverted orientations, interchromosomal changes, amplification, and deletions [404]. The *TERT* gene can also be a target of this alteration. Unlike other TMMs, rearrangements have only been extensively explored, to our knowledge, in a single tumour subset: neuroblastoma (NBL). NBL is a malignant embryonic tumour that arises from the peripheral sympathetic nerve system and represents the most common extracranial solid tumour in children associated with unfavourable patient outcome [339,340,405].

Amplification of the proto-oncogene *MYCN* has been used for many years in these patients as a reliable marker for defining high-risk disease [406], but only recently, recurrent genomic rearrangements proximal of the *TERT* gene have been reported in NBL, defining a subgroup of high-risk tumours with a particular poorer outcome [54,340]. At the present time, both *TERT* rearrangements [54,339,340] and *MYCN* amplification [339,340,341] are two well-established indicators of poor prognosis. The most aggressive form of the disease appears to be related with high telomerase activity [339,340,407], which is caused by both alterations [54,408,409,410]. Some studies reported that structural rearrangements affecting the chromosomal region at 5p15.33 lead to juxtaposition of strong enhancer elements in close proximity to the *TERT* locus [45,54,339,341], resulting in a massive transcriptional upregulation of TERT and adjacent genes distal of the breakpoints, and a strong epigenetic remodelling of the affected region (histone modifications and DNA methylation) [54,339,341]. Epigenetic marks were reported as absent in NBLs without these rearrangements [54]. These rearrangements occurred only in high-risk NBLs [54,339] in a mutually exclusive fashion with *MYCN* amplifications and *ATRX* mutations [54] that are common genetic alteration in NBLs [340]. *TERT* rearrangements are structurally diverse [54], as translocations are both inter and intrachromosomal [405]. Low-risk NBLs lack evidence of active TMMs and high-risk NBLs without *TERT* or *MYCN* alterations lack telomerase activity and are characterized by activation of the ALT pathway [339]. 

Moreover, intratumoural diversity in TL is another feature in NBL [341]. The diversity of TL in individual NBLs was strongly associated with disease progression and death [317,411,412]. On the basis of these studies, Jeison et al. [412] defined two subtypes of NBLs with poor clinical outcome: the first comprising cases with *MYCN* amplification, typically demonstrating decreased or unaltered TL, and the second comprising cases presenting normal MYCN status and increased TL. When combining high-risk and high-stage NBLs, *TERT* rearrangements account for 18% of the cases, in contrast to the 13% observed in low to high-stages NBLs [54,340,405]. 

*TERT* rearrangements in NBLs represent the second most frequent genetic defect following *MYCN* alterations. In NBLs, the *TERT* rearrangements were almost mutually exclusive with *MYCN* and *ATRX* (associated to another TMM, ALT), stratifying them in two groups of NBLs with very high risk, reinforcing the concept that tumours do not present multiple TMM simultaneously. 

#### 2.2.5. Epigenetic Mechanisms

Epigenetic alterations consist of alterations that affect cell behaviour through events other than direct DNA sequence changes, as the ones previously described [413,414]. Rather, these modifications regulate patterns of gene expression by modifying DNA accessibility by transcription factors and chromatin structure [414,415]. These biochemical pathways are crucial to normal development and differentiation of distinct cell lineages in the adult organism, rendering epigenetic mechanisms (EMs) an important tumorigenic effect [413,414,415]. 

Noteworthy, lifestyle changes influence epigenetic regulation of TR [416]. For instance, chronic psychological stress is believed to contribute to telomere shortening in humans at different life stages [417,418] in an apparent dose-dependent way [419], and diverse dietary compounds can have an impact on *TERT* by modulating the activity of DNA methyltransferases (DNMTs) and histone-modifying enzymes [420,421].

Nowadays, the most studied epigenetic alterations associated with neoplastic phenotype include DNA methylation and micro RNA (miRNA) mediated targeting of various genes [413,414]. Reactivation of telomerase is controlled by these mechanisms [420].

DNA methylation was the first identified EM [415,420]. This epigenetic process is crucial in gene expression, and errors in its pattern are tightly related to tumour initiation and progression [31,415,420]. Generally, via DNMTs, DNA methylation occurs genome-wide in non-coding regions, at CpG sites, which occur densely in regions known as CpG islands [31,415,422]. These CpG islands are located within gene promoter regions of approximately 60–70% of human genes and facilitate their expression by enabling the interaction with transcription factors [415,420]. 

Tumour cells can take advantage of two patterns of DNA methylation [415]: hypermethylation—increased methylation of CpG islands, generally associated with gene silencing of tumour suppressors such as *p16* [423], *MLH1* [424,425], and *MGMT* [72]—and hypomethylation—an overall decrease in global DNA methylation pattern, typically associated with overexpression of proto-oncogenes and growth factors [426].

Paradoxically, other genes, such as *TERT*, represent an exception to this regulation, as some authors reported that *TERTp* hypermethylation correlates with TERT overexpression in telomerase-positive cells [72,415,427,428], while the absence of *TERT* methylation was reported in some TERT-negative tumours and TERT-negative normal cells [28,72,429]. These findings are very dependent on the tissue of origin, since some authors did not find any correlation between mean methylation levels or hypermethylation and TERT expression levels in sporadic gastric adenocarcinoma, NOS [430], various histotypes of cervical (adenocarcinoma, SCC, adenosquamous, and carcinofibroma) [431] and ovarian (serous and cystoadenomas, endometrioid, and clear cell) [431,432] carcinomas; in gliomas (including WHO grades II–IV astrocytic and WHO grades II–III oligodendroglial tumours) some results are contradictory [72]. It seems that *TERTp* methylation is essential for TERT expression, and thus telomerase activity [420]. Indeed, in most TERT-positive tumour cells, most of the *TERTp* region contains hypermethylated CpG islands [420]. 

The degree at which the *TERT* promoter is methylated plays a role in carcinogenesis [420]. Hypermethylated states can prevent transcriptional repressors [433], such as CTCF [434], SIN3A, or MAZ [435] from binding to the target DNA-binding sites in the region. Lower methylation level allows the linkage of transcriptional repressors, resulting in shorter telomeres and lower telomerase activity [436]. Downregulation of DNMTs, which reduces the hypermethylated state of the *TERTp* by allowing repressor binding, is the proposed mechanism for telomerase reactivation [45,420]. 

There is a strong association between *TERT* methylation and telomerase activity in some tumour types, including B-cell lymphocytic leukaemia [437], colorectal carcinoma, NOS [438] and pancreatic ductal adenocarcinoma [439]. Colorectal carcinoma displays a higher level of cell methylation within the *TERT* promoter and high degree tumours with TERTp methylation reveal high telomerase activity [440]. Both DNA methylation and histone modification seem to operate TERT regulation in HCC [441]. Fan et al. [433] reported that *TERTp* CpG methylation may represent an alternative pathway to *TERTp* mutations in cutaneous melanoma, NOS. 

Overall, the most characterized *TERTp* hypermethylation at specific CpG islands, also named as THOR (*TERT* hypermethylated oncological region), has been reported to have diagnostic and prognostic value in prostate, NOS [442] and pancreatic (exocrine and endocrine, NOS) cancers [443]. Methylation of the *TERTp* has also been suggested as a biomarker for malignancy and patient outcome in paediatric gliomas, such as pilocytic astrocytoma, medulloblastoma, NOS, ependymoma, choroid plexus carcinoma, among others [338].

Specific non-coding RNA interaction with *TERT* has been reported in multiple types of tumours [420]. TERT regulation by miRNA was summarized by Lewis et al. [420]. It was proposed that miRNAs can post-transcriptionally alter *TERT* transcripts directly or indirectly, affecting regulatory transcription factors [5,420]. TERRA transcripts are other types of non-coding RNAs found in eukaryotes that will be further addressed due to their association in ALT.

#### 2.2.6. Alternative Lengthening of Telomeres 

**Figure 2:**
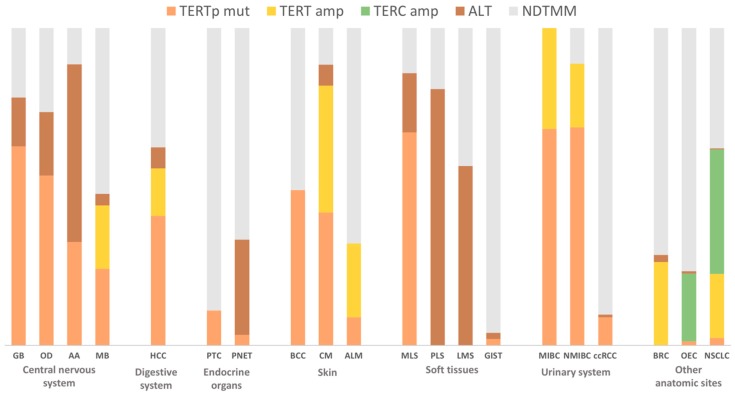
Frequency of telomere maintenance mechanisms in tumours by organ/anatomical site. The rates presented correspond to tumour types in which the following mechanisms were studied: *TERTp* mutations (*TERTp* mut) (orange), *TERT* amplification (*TERT* amp) (yellow), *TERC* amplification (*TERC* amp) (green), and ALT (brown). The studied population for each TMM is composed of the following cohorts (when not specified the data is depicted in Table 1, Table 2 and Table 4): Glioblastoma (GB), NOS (*TERTp* mut, *n* = 523; ALT, *n* = 953); oligodendroglioma (OD), NOS (*TERTp* mut, *n* = 469; ALT, *n* = 40); anaplastic astrocytoma (AA), NOS (*TERTp* mut, *n* = 89; ALT, *n* = 143); medulloblastoma (MB), NOS (*TERTp* mut, *n* = 166; TERT amp, *n* = 15; ALT, *n* = 192); hepatocellular carcinoma (HCC) (*TERTp* mut, *n* = 3091; *TERT* amp, *n* = 181; ALT, *n* = 121); papillary thyroid carcinoma (PTC), NOS (*TERTp* mut, *n* = 3256); pancreatic neuroendocrine tumour, NOS (pNET) (*TERTp* mut, *n* = 123; ALT, *n* = 849); basal cell carcinoma (BCC) (*TERTp* mut, *n* = 292); cutaneous melanoma, NOS (CM) (*TERTp* mut, *n* = 1975; *TERT* amp, *n* = 10; ALT, *n* = 106); acral lentiginous melanoma (ALM) (*TERTp* mut, *n* = 452; *TERT* amp, *n* = 60); myxoid liposarcoma (MLS) (*TERTp* mut, *n* = 76; ALT, *n* = 107); pleomorphic liposarcoma (PLS) (ALT, *n* = 26); leiomyosarcoma (LMS) (ALT, *n* = 161); gastrointestinal stromal tumour (GIST) (*TERTp* mut, *n* = 251; ALT, *n* = 50); muscle invasive bladder carcinoma (MIBC) (*TERTp* mut, *n* = 679; *TERT* amp, *n* = 3; ALT, *n* = 150); non-muscle invasive bladder carcinoma (NMIBC) (*TERTp* mut, *n* = 1682; *TERT* amp, *n* = 15); clear cell renal cell carcinoma (ccRCC) (*TERTp* mut, *n* = 443; ALT, *n* = 117); breast carcinoma (BRC) (*TERT* amp, *n* = 19; ALT, *n* = 377); oesophageal carcinoma (OEC) (*TERTp* mut, *n* = 403; *TERC* amp, *n* = 168; ALT, *n* = 136); non-small cell lung cancer (NSCLC) (*TERTp* mut, *n* = 961; *TERT* amp, *n* = 769; *TERC* amp, *n* = 176; ALT, *n* = 353). We assume for the percentage in which no defined TMM was reported that a non-defined telomere maintenance mechanisms (NDTMM) may be operating (grey).

About 10 to 15% of tumours achieve immortalization through a telomerase-independent mechanism of telomere lengthening—alternative lengthening of telomeres [34], which was first detected in a telomerase-null mutant yeast [444] and subsequently reported in human tumours and tumour-derived cell lines [35,445]. ALT-positive cells are dependent on the activation of a homologous recombination DNA-repair mechanism to maintain telomere length. The knowledge regarding this mechanism has grown gradually throughout the years [34,444,445,446,447]. These cells are characterized by specific phenotypic features, such as heterogeneous telomere lengths [448,449], ALT-associated promyelocytic bodies (APBs) with decreased telomeric repeat binding factor 2 (TRF2) density [450,451], and telomere recombination with the presence of extrachromosomal (linear and circular) telomeric repeats [452]. Telomere-specific FISH (tel-FISH), APBs immunofluorescence and ALT-associated molecules (mainly C-circles) detection assays are some of the most commonly used techniques to detect ALT; tel-FISH and APBs immunofluorescence can be used in combination [453,454]. When reporting ALT phenotype (Table 4 and Figure 2), it should be kept in mind that, due to the variety of methodologies used, the positivity threshold may vary among studies.

Tumours of mesenchymal origin are reported to activate ALT more frequently, which may be explained by the fact that mesenchymal stem cells express minimal or undetectable telomerase, i.e., their lineage seems dependent on the activation of an alternative mechanism to maintain TL [55,453]. Recent studies reported that mutations in the *ATRX* or *DAXX* genes that encode chromatin remodelling proteins essential for the deposition of the histone variant H3.3 at telomeric and pericentromeric regions of the genome influence the activation and maintenance of ALT in pNETs [313,455], paediatric glioblastoma [337], and a spectrum of other tumours [55,321,322]. *ATRX*/*DAXX* mutations are thus suggested to be strong contributors to the activation of the ALT pathway. New evidence of epigenetic mechanisms affecting ALT were recently reported, such as microRNA regulation, as reviewed by Naderlinger et al. [5]. This mechanism has also been reported to correlate with high levels of TERRA [456].

The prevalence of ALT phenotype in tumour subtypes is shown in Table 4. ALT presents a high frequency in CNS tumours, such as diffuse and anaplastic astrocytomas, NOS (52% and 44%, respectively), adult and paediatric forms of glioblastoma (15% and 30%), among other types. The presence of ALT mechanism in GBs identifies less aggressive tumours with a longer patient survival, being associated at the same type to younger patients [298,301,303]. At variance, NBLs showing telomere elongation by this mechanism (50%) are characterized by unfavourable prognosis and resistance to chemotherapy [342]. Regarding ATRX status, high-grade astrocytomas have been reported with concomitant ALT phenotype and ATRX loss, in both adult and paediatric tumours [295,296,298,337]. Such findings point towards a central role of ATRX in ALT in CNS tumours [337]. A recent study by Fogli et al. [302] reported the presence of ALT in high-grade gliomas associated with *IDH1*/2 mutation, O-6-Methylguanine-DNA Methyltransferase (MGMT) methylation, absence of functional ATRX protein and elevated TERRA levels, supporting the need for more studies in these tumours, since their molecular background seems to have major importance in the stratification of patient prognosis.

Various subtypes of tumours that are reported with high frequencies of *TERTp* mutations, as previously addressed, do not present ALT. 

ALT phenotype has also been reported to be highly prevalent in a wide variety of soft tissue and bone tumours: dedifferentiated liposarcoma (33%), pleomorphic liposarcoma (80%), myxofibrosarcoma (76%), leiomyosarcoma (57%), osteosarcoma (63%), malignant peripheral nerve sheath tumours (63%), among others. It has been associated with worse patient prognosis in some of these tumours [319,320,329], and it has been also correlated with loss of ATRX expression [322]. Bone and soft tissue sarcomas show a relatively lower or even absent frequency of *TERTp* mutations, with the exception of myxoid liposarcoma, that presents a high prevalence of *TERTp* mutations (67%) and a lower prevalence of ALT (15%). 

Pancreatic neuroendocrine tumours are also reported to activate ALT frequently (30%). In this type of tumours, a consistent correlation between ALT phenotype and inactivation of either *ATRX* or *DAXX* has been reported [309,311,312,314]; however, ALT-positive cases that preserve expression of ATRX and DAXX indicates the presence of other activators [5]. ALT is also suggested to predict metastatic disease and poor survival in these tumours [306,309,311,312,334].

Overall, ALT phenotype is extremely rare in carcinomas; it has been described in certain subtypes such as ductal breast carcinoma (2%), HCC (7%), clear cell carcinoma, and endometrioid carcinoma of the ovary (4% and 1%, respectively) and chromophobe and sarcomatoid carcinomas of the kidney (9% and 7%, respectively). It is also present in malignant melanoma, NOS (7%), small cell neuroendocrine carcinoma of the bladder (23%), and in medullary thyroid carcinoma (26%). Omori et al. [305] reported a prevalence of 38% in gastric adenocarcinoma, NOS, but a subsequent study with a higher number of cases reported a negative ALT phenotype in such tumours [55]. 

Noteworthy, ALT was observed not to be present in several benign tumours of different origins, namely colon, hepatocellular, thyroid, and parathyroid adenomas [55]. There are still a lot of unanswered questions about these ALT mechanisms, mainly the ones regarding the molecular basis of its activation in tumour cells with wildtype *ATRX* or *DAXX*. The tumours with higher prevalence of ALT are reported as the least *TERTp*-mutated types, with the exception of gliomas (including WHO grades II–IV diffuse astrocytic and oligodendroglial tumours), in which a high frequency is observed for both mechanisms of cell immortalization. As illustrated in Figure 2, regarding tumours from different origins, the landscape of the distribution of TMMs by organ/anatomical site is quite diverse and with different cumulative prevalence. Evidences regarding mutual exclusivity of *TERTp* mutations and ALT phenotype in several types of tumours point towards the fact that when cells do not rely on telomerase activation to achieve immortalization, they activate the ALT mechanism. Some studies reported concomitant TERT expression and ALT activation (in adrenocortical carcinoma [308], NBL [317], osteosarcoma [327], nephroblastoma [330]), without clarifying the mechanism underlying telomerase reactivation. A recent study by Hayward et al. [187] has reported unexpected findings in a subset of cutaneous melanomas, in which nine in 10 *ATRX*-mutated cases also presented *TERTp* mutations, but these are novel findings that require further clarification.

#### 2.2.7. Non-Defined Telomere Maintenance Mechanism 

Data from TMM analyses gathered in the last years unveiled a phenotype in which both telomerase (or TERT) expression and ALT were reported as absent, pointing to a novel TMM: the non-defined telomere maintenance mechanism (NDTMM). Glioblastoma [303,457,458], osteosarcoma [328,459], metastases of cutaneous melanoma [460,461], and other tumour types [35,45] presented such a phenotype. Interestingly, Royds et al. [462] reported a NDTMM in GBs as a distinctive phenotype characterized by reduced patient survival, association with a polymorphism in *CDKN2A* and rarely *IDH1*-mutated. Analysing all the information previously described in this review, we recognized that some of the most incident cancers worldwide do not present any reported TMM (Table 6), what could be due to a failure in detection or, alternatively, represent a NDTMM. Noteworthy, the NDTMM frequencies reported in Figure 2 were obtained assuming the reported TMMs as mutually exclusive, what, as aforementioned, may not be transversal to all tumour cases. For this reason, and since most studies aim to assess a single TMM, the frequencies here reported are most likely underestimated. Nonetheless, these results represent a large proportion in several tumour types that must not be neglected. Tumours harbouring a NDTMM do not always present the same telomeric features [45], raising the question of which mechanism(s) is behind the maintenance or even if one exists, warranting the need for more studies on the matter. TERRA molecules may play a role in NDTMM. These are nuclear long noncoding RNAs (lncRNAs) found in all eukaryotes [5,37,463,464] that contain subtelomeric and telomeric UUAGGG-repeats transcribed by RNA polymerase II from the subtelomere towards the telomere [463,465,466]. They can regulate genome function by recruiting chromatin modifiers, regulating protein activity as trans-acting factors, and performing architectural functions [39,467]. TERRAs are also proposed to bind the telomerase core components, *TERT* and *TERC* [468], with stronger affinity for the later [469]. TERRA appears to integrate all lncRNAs functions into a single transcript responsible for telomere maintenance regulation in response to cellular signals [39].

Overall, TERRA molecules have been implicated in: (1) heterochromatin formation [470,471]; (2) direct inhibition of telomerase, by potential competition with the telomeric substrate for telomerase interactions [37,463,466,472]; (3) telomere protection [470,473]; (4) telomere replication in altered ALT cells lacking ATRX [474]; (5) telomere elongation by HR through the formation of DNA-TERRA hybrids [475]; and (6) participation in DDR activated by dysfunctional telomeres [471]. 

Naderlinger et al. [5] pointed to the enticing rational distinction between the potential use of these multi-featured molecules as templates for a new mechanism of telomeric synthesis. Luke et al. [464] suggested that instead of an essential or a permanent constituent of the telomeric chromatin, TERRA may have a transient regulatory role depending on telomeres’ specific functional state, by detecting TERRA molecules by RNA-FISH only in a subset of telomeres at human and mouse chromosome ends. Also, Rippe et al. [39] proposed that TERRA functions might be regulated in a telomere state-dependent manner because different telomere states may result in altered access of TERRA regulators to different telomere types: (a) at normal-length telomeres, TERRA appears to inhibit its own expression through EMs, by recruiting factors that promote a repressive chromatin state via the transcription-silencing network played by histone methyltransferase SUV39H, trimethylated H3K9 histone (H3K9me3) (essential for telomeres that use telomerase as a TMM), and heterochromatin proteins HP1 [5,37,39,476]; (b) when telomeres are shortened or damaged, TERRA levels increase, possibly due in part to their inability to play TPE-OLD, and also to a deactivation of autorepressive mechanisms (decrease of H3K9me3 levels [476] or depletion of TRF2 [476,477], a DNA-binding sheltering subunit) [39,476,478]; (c) when ALT pathway is responsible for telomere maintenance, TERRA expression levels appears more highly expressed than telomerase-positive cells [37,39,479]. It was proposed that the association of TERRA with telomeres in ALT cells is controlled by an interlinked network of TERRA, ATRX, H3K9me3, and TRF2 [39]. As already discussed, ALT cells highly express TERRA [456] and have loss of functional ATRX and incorporation of the histone H3.3 [480]. Consequently, H3K9me3 heterochromatin modification may decrease and ALT-associated decrease density of TRF2 [451] may contribute to raising TERRA levels, by relieving the TRF2-depedent TERRA silencing network [39]. ATRX depletion may stabilize TERRA’s association with telomeres, thus leading to eventual replication stress and increased replication-fork stalling [39]. Still, no clear correlation between ATRX and global TERRA expression levels is apparent [475,479,480]. Low TERRA levels, in combination with low to absent TR, were tentatively associated with favourable patient prognosis in a cohort of patients with grade II–IV astrocytomas [456].

## 3. Final Remarks

This extensive data collection allowed us to characterize the current panorama of TMMs in human cancers, in what regards to their prevalence, association to histopathological and molecular tumour features, prognostic assessment, and impact on clinical management.

*TERTp* mutations are the most frequent somatic non-coding alterations harboured by a wide spectrum of human tumours, namely of CNS, thyroid, skin, bladder, and liver. The collected data disclose remarkable differences of prevalence of *TERTp* mutations in histotypes from the same organs, as well as different TMMs within the same histotypes. The reason(s) for such differences remains unclear. One of the most important results concerning *TERTp* mutations are their frequent association to worse prognostic features and poorer patient survival. This finding indicates that *TERTp* mutations may be used as a biomarker for patient stratification in some cancers. Such mutations can arise in the context of malignant transformation in certain histotypes (e.g., liver), but overall, they represent a late event in most cancers. In tumours arising from tissues that are highly exposed to environmental factors (e.g., skin and bladder), *TERTp* mutations represent an early event. At variance with this aforementioned influence of *TERTp* mutations in many human cancers, there are some histotypes that do not present such alterations. The absence of selection for *TERTp* mutations can be partially explained by the fact that such tumours occur in tissues with fast cellular renewal, such as gastrointestinal or haematological malignancies. In the later context, the telomere length needs to be regularly maintained and may present intrinsic telomerase activity, making the existence of an activating telomere maintenance mechanism less important as a means for providing an additional selective advantage to cancer cells. The collected data show that for some tumours, *TERTp* mutations can be additionally modulated by *TERTp* germline genetic variations. Actually, such SNPs have been reported to impact the prognosis of *TERTp*-mutated tumours (e.g., urothelial bladder carcinomas and glioblastomas). The SNPs that modulate TERT transcriptional capacity are not restricted to the *TERTp*: as GWA studies have demonstrated, there are *TERT* germline genetic variations that lead to an increased risk of developing several cancer types. Since the results are sometimes conflicting with regard to the same histotypes, the interpretation of the impact of such polymorphisms must be carefully balanced, taking into consideration population (or ethnic) disparities and, ultimately, tumour genetic backgrounds.

*TERT* and *TERC* amplifications and *TERT* rearrangements were found in a small percentage of the reviewed cases. The scarce knowledge about these mechanisms determines the need for studying larger series in order to evaluate the real impact and frequency of such findings. The data obtained up to now provide promising evidence to be used as a diagnostic and prognostic tool in uterine malignancies and neuroblastomas, respectively, a feature that may be incorporated in the future clinical practice. 

As previously indicated, ALT is highly prevalent in tumours of mesenchymal origin (e.g., soft tissues and bone tumours). Striking exceptions were grades II to IV diffuse astrocytic and oligodendroglial tumours, which are prone to exhibit *TERTp* mutations and ALT, usually displaying mutual exclusivity. Both alterations aid in the prognostic stratification of the observed patients. The aforementioned tumour types are the best examples of the role played by telomere status on prognosis using multiple maintenance mechanisms. *ATRX* inactivating mutations are intrinsically linked to ALT in CNS tumours and other tumour histotypes (e.g., pNETs). However, in *ATRX*-wildtype tumours, the mechanisms underlying ALT activation remain to be elucidated. Until recently, there was a mutual exclusivity of *TERTp* mutations and ALT activation, but recent studies reported their concomitant presence. Ongoing studies evaluating tumour inter- and intra-heterogeneity will be important to clarify the aforesaid category to find if subclones within a tumour or even if the same cell may at some point harbour both mechanisms simultaneously and possibly select one of them afterwards. 

It was noteworthy that tumours of the breast, stomach, small intestine, colon and rectum, exocrine pancreas, lung, and prostate, that represent some of the most frequent tumours worldwide, were also those that presented a low frequency or absence of known TMMs. Breast and colorectal tumours were found to have a high prevalence of *TERT* amplifications, although the respective cohorts were too small to solely assign this mechanism to telomere maintenance. In the study of Barthel et al. [45], 22% of the cases had no detectable TERT expression nor alterations in the genes directly linked to ALT activation—*ATRX* and *DAXX*. Barthel et al. [45] hypothesized that not all tumours harbour immortalized cells with a TMM or that additional mechanisms may yet exist, ‘something’ we may designate as a non-defined TMM. Such (yet) undefined TMM may involve *RB1* or *TP53* alterations due to telomere-driven genomic instability, that may surpass the DNA repair mechanisms [45].

Finally, we have emphasized throughout the text the intriguing questions that remain to be answered, such as the reasons behind the (a) gradual increase in TMM activation with grade progression; (b) high-grade dependence of some histotypes for specific TMMs; (c) the homogenous distribution of TMMs frequencies among very different tumour grades; (d) better prognosis conferred by TMMs in exceptional cases, and, at last; for the (e) apparent absence of TMMs in some tumours. Pursuing these questions will open new avenues in the understanding of mechanisms that may surpass the classical TMMs function but with the same end-result—to assure cancer cell immortality. 

## Figures and Tables

**Table 1:** Prevalence of *TERTp* mutations in human tumours. Only the tumour histotypes associated with a frequency of *TERTp* mutations ≥5% appear together with the respective number, percentage, and range of mutated cases. Whenever the tumour histotypes were associated with a low rate of *TERTp* mutations (<5%) only the total of patients is shown (complete data available in Supplementary Table S1). The designations ‘NOS’ (not otherwise specified) and ‘SNS’ (site not specified) were applied to the tumours in which a specific histotype/site was not available.

Tumour Type	Mutated Cases (Total)	Mutated Cases in % (Range)
**Tumours of the breast**		
Fibroadenoma [58]	4 (58)	6.9
Phyllodes tumour [58,59]	41 (70)	58.6 (45.8–65.2)
<5%: breast carcinoma, NOS (*n* = 88) and breast cancer, NOS (*n* = 122) [49,60].		
**Tumours of the central nervous system**		
Astrocytoma (grades II-III), NOS [61,62,63]	160 (699)	22.9 (18.2–39.3)
Primary glioblastoma, NOS [50,64,65,66,67,68,69,70]	1216 (1800)	67.6 (46.3–85.9)
Secondary glioblastoma, NOS [50,64,65,67]	25 (99)	25.3 (5.0–35.7)
Paediatric glioblastoma, NOS [49,50]	3 (51)	5.9 (3.1–10.5)
Diffuse astrocytoma *IDH*-mutant [50,64,71,72,73]	38 (242)	15.7 (7.7–31.6)
Diffuse astrocytoma *IDH*-wildtype [50,64,71,72,73]	23 (87)	26.4 (7.1–58.3)
Diffuse astrocytoma, NOS [67,74,75,76,77]	112 (574)	19.5 (7.7–32.0)
Anaplastic astrocytoma, *IDH*-mutant [50,64,71,73,78,79]	28 (248)	11.3 (4.4–20.0)
Anaplastic astrocytoma, *IDH*-wildtype [50,64,71,73,78,79]	104 (220)	47.3 (20.0–80.0)
Anaplastic astrocytoma, NOS [67,74,75,76]	29 (89)	32.6 (10.0–40.0)
Glioblastoma, *IDH*-wildtype [61,62,63,64,67,68,71,74,77,78,79,80,81,82]	2082 (2899)	71.8 (27.6–91.7)
Giant cell [50]	6 (17)	35.3
Gliosarcoma [50]	21 (26)	80.8
Glioblastoma, *IDH*-mutant [61,62,63,64,67,68,71,74,77,78,79,80,81]	114 (470)	24.3 (0.0–76.9)
Glioblastoma, NOS ^a^ [66,75,76,83]	328 (523)	62.7 (46.3–84.0)
Oligodendroglioma, *IDH*-mutant and 1p/19q-codeleted [50,63]	296 (311)	95.2
Oligodendroglioma, NOS [49,62,64,67,68,73,74,75,77]	251 (469)	53.5 (7.7–75.7)
Anaplastic oligodendroglioma, *IDH*-mutant and 1p/19q-codeleted [50]	8 (38)	21.1
Anaplastic oligodendroglioma, NOS [49,50,64,67,73,74,76]	171 (273)	62.6 (0.0–88.5)
Oligodendroglioma (grades II–III), NOS [61,78]	132 (152)	86.8 (79.3–96.9)
Oligoastrocytoma, NOS ^b^ [49,50,61,62,64,67,73,74,76]	222 (618)	35.9 (3.8–57.9)
Anaplastic oligoastrocytoma, NOS [49,50,64,67,73,74,77]	170 (415)	41.0 (26.7–52.3)
Ependymoma ^c^ [49,50]	6 (118)	5.1 (0.0–6.9)
Choroid plexus carcinoma [50]	1 (6)	16.7
Dysembryoplastic neuroepithelial tumour [49,50]	1 (15)	6.7 (0.0–33.3)
Desmoplastic infantile astrocytoma and ganglioglioma [50]	1 (8)	12.5
Paraganglioma [50]	1 (12)	8.3
Medulloblastoma, adult (>18 y) WNT-activated [50,84]	5 (15)	33.3 (30.8–50.0)
Medulloblastoma, adult (>18 y) SHH-MB [50,84,85,86]	119 (134)	88.8 (72.7–100.0)
Medulloblastoma, adult (>18 y), NOS [50]	15 (23)	65.2
Medulloblastoma, paediatric SHH-MB ^d^ [50,84,86]	49 (146)	33.6 (20.0–31.9)
Medulloblastoma, paediatric, NOS [50,84,86]	22 (121)	18.2 (3.5–56.0)
Medulloblastoma, NOS [49,75,87]	40 (166)	24.1 (20.9–33.3)
Meningioma with malignant histology [88]	5 (18)	27.8
<5%: pilocytic astrocytoma (*n* = 167) [50,75,76], pilomyxoid astrocytoma (*n* = 3) [50], subependymal giant cell astrocytoma (*n* = 11) [50,75], pleomorphic xanthoastrocytoma (*n* = 26) [50,75], mixopapillary ependymoma (*n* = 14) [50], subependymoma (*n* = 12) [50], anaplastic ependymoma (*n* = 48) [50], angiocentric glioma (*n* = 9) [50], choroid plexus papiloma (*n* = 13) [50], gangliocytoma (*n* = 2) [50], ganglioglioma (*n* = 40) [50], anaplastic ganglioglioma (*n* = 3) [50], papillary glioneuronal tumour (*n* = 1) [50], rosette-forming glioneuronal tumour (*n* = 6) [50], central neurocytoma (*n* = 28) [50], pineocytoma (*n* = 2) [50], pineal parenchymal tumours of intermediate differentiation (*n* = 9) [51], pineoblastoma (*n* = 5) [50], papillary tumours of the pineal region (*n* = 2) [50]), medulloblastoma, paediatric WNT-activated (*n* = 66) [50,84,86] and meningioma (*n* = 349) [49,75,89].		
**Tumours of the conjunctiva and uvea**		
Primary acquired melanosis with atypia [90]	2 (25)	8.0
Conjunctival melanoma [90,91,92]	32 (84)	38.1 (31.6–57.1)
Perilimbal squamous carcinoma [93]	21 (48)	43.8
<5%: primary acquired melanosis without atypia (*n* = 13) [90], conjunctival naevi (*n* = 56) [90] and uveal melanomas (*n* = 202) [90,91,92,94].		
**Tumours of the digestive system**		
Borderline hepatocellular adenoma/hepatocellular carcinoma [95]	3 (18)	16.7
Hepatocellular carcinoma derived from hepatocellular adenoma [95,96]	12 (25)	48.0 (43.8–55.6)
Fibrolamellar hepatocellular carcinoma [49,97]	1 (20)	5.0 (0.0–12.5)
Hepatocellular carcinomas ^e^ [45,49,75,96,97,98,99,100,101,102,103,104,105,106,107,108,109]	1263 (3093)	40.8 (26.3–63.3)
Gallbladder carcinoma, NOS [49,75,110]	15 (166)	9.0 (0.0–50.0)
<5%: oesophageal squamous cell carcinoma (*n* = 313) [111], oesophageal adenocarcinoma, NOS (*n* = 90) [112], gastric moderately differentiated adenocarcinoma, NOS (*n* = 29) [113], moderately to poorly differentiated gastric adenocarcinoma, NOS (*n* = 39) [113], poorly differentiated gastric adenocarcinoma, NOS (*n* = 119) [113], gastric mucous adenocarcinoma (*n* = 13) [113], well-differentiated gastric cancer, NOS (*n* = 90) [110], poorly-differentiated gastric cancer, NOS (*n* = 178) [110], gastric carcinomas of both intestinal and diffuse types, NOS (*n* = 74) [75], colorectal adenocarcinoma, NOS (*n* = 22) [49], hepatoblastoma (*n* = 18) [49,114], hepatocellular adenoma, NOS (*n* = 238) [95,97], intrahepatic cholangiocarcinoma (*n* = 65) [97,99]), extrahepatic cholangiocarcinoma (*n* = 6) [75], extrahepatic cholangiosarcoma (*n* = 28) [49], pancreatic ductal adenocarcinoma (*n* = 24) [49], pancreatic acinar cell carcinoma (*n* = 25) [49] and pancreatic cancer, NOS (*n* = 46) [49].		
**Tumours of the endocrine organs**		
Benign thyroid tumour, atypical follicular adenoma * [115]	3 (18)	16.7
Papillary thyroid carcinoma, conventional variant [51,116,117,118,119,120,121,122,123,124,125]	178 (1838)	9.7 (2.9–15.2)
Papillary thyroid carcinoma, follicular variant [51,116,117,118,120,122,123,124,125]	27 (481)	5.6 (0.0–13.3)
Papillary thyroid carcinoma, tall cell variant [116,117,118,120,124,125,126]	40 (214)	18.7 (0.0–60.0)
Papillary thyroid carcinoma, NOS [51,118,119,120,123,125,127,128,129,130,131,132,133,134,135,136,137,138,139]	361 (3288)	11.0 (0.0–40.0)
Follicular thyroid carcinoma [51,115,116,119,120,122,125,128,129,132,135,138,139]	102 (564)	18.1 (13.9–50.0)
Hürthle cell carcinoma ^f^ [120,127,140]	11 (146)	7.5 (4.8–33.3)
Poorly differentiated thyroid carcinoma [51,116,120,122,125,127,133,135]	88 (217)	40.6 (21.4–51.7)
Anaplastic thyroid carcinoma [51,116,122,125,127,129,133,135,141,142]	150 (326)	46.0 (12.5–81.5)
Metastases of well-differentiated papillary and follicular thyroid carcinomas, NOS [118,125,138,143]	90 (437)	20.6 (15.0–50.0)
Adrenal cortical carcinoma [144]	4 (34)	11.8
Extra-adrenal paraganglioma **^g^** [144]	1 (9)	11.1
<5%: pituitary adenoma (*n* = 15) [145], follicular thyroid adenoma (*n* = 263) [51,115,122,128,139], benign thyroid tumour, NOS (*n* = 44) [146], papillary thyroid carcinoma, hobnail variant (*n* = 10) [147], papillary microcarcinoma (*n* = 435) [148,149], paediatric papillary carcinoma (*n* = 105) [150,151], minimally invasive Hürthle cell tumour (*n* = 52) [127,140], medullary thyroid carcinoma (*n* = 135) [49,51,116,119,129], adrenal cortical adenoma (*n* = 47) [144], phaechromocytoma (*n* = 122) [51,144], extra-adrenal benign paraganglioma * (*n* = 4) [144], pancreatic neuroendocrine tumour, NOS (*n* = 123) [49,57].		
**Tumours of the female reproductive organs**		
Ovarian low-grade serous carcinoma [49,152]	2 (41)	5.0 (3.0–12.5)
Ovarian clear cell carcinoma [49,152,153]	48 (301)	15.9 (15.9–16.7)
Uterine endometrial carcinoma, NOS [49]	2 (19)	10.5
Uterine clear cell carcinoma, NOS [153]	3 (14)	21.4
Cervical squamous cell carcinoma [49,152,154,155]	33 (225)	14.7 (0.0–21.4)
Vulvar melanoma [156]	2 (23)	8.7
<5%: high-grade ovarian serous carcinoma (*n* = 80) [152], ovarian endometrioid carcinoma (*n* = 43) [152], uterine endometrioid carcinoma (*n* = 24) [152], uterine leiomyosarcoma (*n* = 22) [152], uterine serous carcinoma (*n* = 12) [152], cervical adenocarcinoma, NOS (*n* = 25) [152] and vulvar melanoma (*n* = 23) [156].		
**Tumours of the head and neck**		
Laryngeal carcinoma, NOS [157]	64 (235)	27.2
Oral squamous cell carcinoma ^h^ [49,154,155,158]	146 (295)	49.5 (2.4–67.7)
Tongue squamous cell carcinoma [49]	10 (28)	35.7
<5%: olfactory neuroblastoma (*n* = 11) [49] and salivary gland tumours (adenoid cystic carcinoma (*n* = 1) [159], adenocarcinoma, NOS (*n* = 1) [159], salivary duct carcinoma, NOS (*n* = 1) [159], epithelial-myoepithelial carcinoma (*n* = 1) [159], adenosquamous carcinoma (*n* = 1) [159], pleomorphic adenoma (*n* = 19) [159], basal cell adenoma (*n* = 1) [159], Warthin tumour (*n* = 8) [159]).		
**Tumours of haematopoietic and lymphoid tissues**		
Mantle cell lymphoma [160,161]	8 (36)	22.2 (0.0–33.3)
<5%: chronic myeloid leukaemia, NOS (*n* = 6) [49], acute myeloid leukaemia, NOS (*n* = 274) [49,162], B-cell acute lymphoblastic leukaemia, NOS (*n* = 12) [160], chronic lymphocytic leukaemia (*n* = 26) [49,160], marginal zone lymphoma (*n* = 16) [160], lymphoplasmacytic lymphoma (*n* = 7) [160], myeloma/plasmacytoma (*n* = 9) [160], follicular lymphoma (*n* = 13) [160], diffuse large B-cell lymphoma (DLBCL), NOS (*n* = 9) [160], plasmablastic lymphoma (*n* = 7) [160] and Burkitt lymphoma (*n* = 9) [160].		
**Tumours of the lung, pleura and thymus**		
Epithelioid mesothelioma [163]	10 (95)	10.5
Sarcomatoid, desmoplastic, and biphasic mesothelioma [163]	6 (15)	15.2
<5%: lung adenocarcinoma, NOS (*n* = 533) [164,165,166,167], lung squamous cell carcinoma, NOS (*n* = 384) [164,165,166,167,168], lung adenosquamous carcinoma (*n* = 44) [154,164,165], mesothelioma, NOS (*n* = 4) [49], thymoma, NOS (*n* = 47) [75], type C thymic cancer * (*n* = 5) [75] and thymic neuroendocrine atypical carcinoma (*n* = 2) [75].		
**Tumours of the peripheral nervous system**		
Absence of histotypes with a frequency of *TERT* promoter mutations equal or higher than 5%.		
<5%: neuroblastoma, NOS (*n* = 153) [49,169].		
**Tumours of the skin**		
Basal cell carcinoma [170,171,172,173]	143 (292)	49.0 (38.8–81.8)
Squamous cell carcinoma [49,154,170,172,173]	57 (102)	55.9 (20.0–74.1)
Bowen disease [172,173]	2 (13)	15.4 (9.1–50.0)
Superficial spreading melanoma [51,92,171,174,175]	129 (380)	33.9 (23.0–40.9)
Nodular melanoma [51,92,171,174,175]	101 (184)	54.9 (42.9–85.7)
Lentigo maligna [51,171,175]	10 (41)	24.4 (0.0–52.9)
Acral-lentiginous melanoma [51,92,171,174,175,176,177,178,179]	40 (452)	8.9 (0.0–27.0)
Desmoplastic melanoma **^i^** [180,181]	43 (96)	44.8 (22.9–85.0)
Cutaneous melanoma **^j^** [45,51,92,156,171,174,175,176,177,178,179,180,181,182,183,184,185,186,187,188,189,190]	826 (1975)	41.8 (7.1–85.0)
Metastatic melanoma of unknown primary site ^k^ [178,179,182]	36 (73)	49.3 (25.0–66.7)
Metastatic cutaneous melanoma **^l^** [185,188]	168 (221)	76.0 (58.8–81.2)
Metastatic melanoma of other primary locations ^m^ [188]	7 (13)	53.8
Mucosal melanoma, NOS [178,179,182]	21 (211)	10.0 (8.7–13.2)
Atypical fibroxanthoma [191]	25 (27)	92.6
Pleomorphic dermal sarcoma ** [191]	26 (34)	76.5
Merkel cell carcinoma [192,193]	6 (63)	9.5 (6.7–10.4)
<5%: cutaneous naevi (*n* = 9) [51].		
**Tumours of the soft tissues and bone**		
Myxoid liposarcoma [49,53,194]	51 (76)	67.1 (23.1–79.2)
Solitary fibrous tumour [49,53,194,195]	37 (175)	21.1 (12.5–27.7)
Fibrosarcoma [49]	1 (3)	33.3
Malignant peripheral nerve sheath tumours [49,53,194,196]	11 (139)	7.9 (0.0–9.6)
Malignant granular cell tumour [53]	1 (2)	50.0
<5%: lipoma (*n* = 8) [49], atypical lipomatous tumour (*n* = 10) [49], dedifferentiated liposarcoma (*n* = 61) [194], pleomorphic liposarcoma (*n* = 15) [194], well differentiated liposarcoma (*n* = 18) [53], dermatofibrosarcoma protuberans (*n* = 16) [53,194], myxofibrosarcoma (*n* = 33) [49,53,194], low-grade fibromyxoid sarcoma (*n* = 18) [49,194], gastrointestinal leiomyoma (*n* = 5) [75], leiomyosarcoma (*n* = 49) [49,53,194] pleomorphic leiomyosarcoma (*n* = 5) [53], rhabdomyosarcoma, NOS (*n* = 5) [53], embryonal rhabdomyosarcoma (*n* = 8) [49], alveolar rhabdomyosarcoma (*n* = 7) [49], angiosarcoma of soft tissue (*n* = 9) [194], gastrointestinal stromal tumour (*n* = 251) [49,51,75,197], gastric schwannoma (*n* = 1) [75], synovial sarcoma (*n* = 48) [49,53,194], epithelioid sarcoma (*n* = 4) [194], alveolar soft part sarcoma (*n* = 9) [53,194], clear cell sarcoma of soft tissue (*n* = 6) [53,194], extraskeletal myxoid chondrosarcoma (*n* = 12) [49,53,194], chondrosarcoma, NOS (*n* = 21) [49,53], Ewing sarcoma (*n* = 6) [53] and undifferentiated high-grade pleomorphic sarcoma (*n* = 70) [49,50,53].		
**Tumours of the urinary system and male genital organs**		
Clear cell renal cell carcinoma [198,199,200]	39 (443)	8.8 (0.0–12.2)
Chromophobe renal cell carcinoma [51,200]	1 (12)	8.3 (0–12.5)
Urothelial muscle invasive bladder carcinoma [201,202,203,204,205,206,207]	463 (679)	68.2 (48.8–85.2)
Urothelial non-muscle invasive bladder carcinoma [51,201,202,203,204,205,206,208,209]	959 (1395)	68.7 (44.3–85.4)
Urothelial bladder carcinoma [45,49,70,75,204,207,210,211]	377 (591)	63.8 (46.4–84.6)
Squamous cell carcinoma of the bladder [168,211,212]	52 (79)	65.8 (54.7–100.0)
Bladder adenocarcinoma ^n^ [210,213]	4 (54)	7.4 (0.0–28.6)
Papillary urothelial neoplasm of low malignant potential [204,214]	26 (43)	60.5 (28.6–75.9)
Urothelial carcinoma of upper urinary tract ^o^ [49,75,200,201,207,215,216]	146 (293)	49.8 (35.3–76.9)
Urothelial carcinoma of the ureter [200,214,215]	26 (135)	19.3 (11.1–50.0)
Micropapillary urothelial carcinoma ^p^ [217]	33 (33)	100.0
Urothelial carcinoma ^q^ [154,218]	71 (96)	75.9 (70.0–100)
<5%: papillary renal cell carcinoma (*n* = 10) [51], nephrogenic adenoma (*n* = 10) [210], prostate squamous cell carcinoma (*n* = 26) [219], prostate carcinoma, NOS (*n* = 47) [49,201], ‘testicular carcinoma’ ***, NOS (*n* = 17) [201].		

^a^ includes cerebellar glioblastoma (*n* = 14) [50], gliomatosis (*n* = 10) [50], glioblastoma with oligodendroglial differentiation (*n* = 6) [50]; **^b^** includes oligoastrocytoma (grades II-III) (*n* = 58) [61]; **^c^** includes spinal ependymoma (*n* = 9); **^d^** these cases were not analysed for the *TP53* status. There is a partial overlap of the study populations of Eckel-Passow et al. [71] and Pekmezci et al. [63]; ^e^ includes grade I-IV hepatocellular carcinoma and clear cell (*n* = 57) [107] and non-clear cell hepatocellular carcinoma, NOS (*n* = 259) [107]; ^f^ includes widely invasive (*n* = 126) [140] and minimaly invasive (*n* = 44) [140] Hürthle cell carcinomas; ^g^ these cases were classified according to the AFIP criteria [220]. It does not seem to exist an overlap of the study populations of Landa et al. [127] and Landa et al. [133]; it may exist an overlap of the study populations of Kim et al. [136] and Kim et al. [137] and the study populations of Melo et al. [125] and Melo et al. [138]; ^h^ includes buccal (*n* = 84) [158], gum (*n* = 34) [158], lip (*n* = 6) [158], tongue (*n* = 63) [158], floor of mouth (*n* = 22) [49,158], alveolar ridge (*n* = 1) [49], mandibule (*n* = 1) [49], hard palate (*n* = 2) [49], supraglottis (*n* = 4) [49], glottis (*n* = 1) [49], tonsil (*n* = 18) [49], larynx (*n* = 2) [49], oropharynx/hypopharynx (*n* = 1) [49] and hypopharynx (*n* = 1) [49]; ^i^ includes pure (*n* = 48) [49] and mixed (*n* = 28) [180] desmoplastic melanoma; ^j^ includes cutaneous melanomas, NOS and spitzoid melanocytic neoplasms (*n* = 56) [183], occult melanoma (*n* = 34), chronically sun-damaged (CSD) (*n* = 18) [179] and non-CSD melanomas (*n* = 12) [179]; ^k^ includes brain (*n* = 11) [182], skin (*n* = 9) [182], bone (*n* = 9) [182], liver (*n* = 13) [182], lung (*n* = 9) [182], visceral lymph nodes (*n* = 16) [182] metastases, and other SNS (site not-specified) metastases (*n* = 34) [178,179]; ^l^ includes superficial spreading melanoma (*n* = 100) [185], nodular melanoma (*n* = 56) [185], lentigo maligna (*n* = 1) [185] and other non-specified metastases (*n* = 156) [185,186]; ^m^ includes lymph node (*n* = 6) [188], brain (*n* = 3) [188], soft tissues (*n* = 2) [188], lung (*n* = 1) [188] and liver (*n* = 1) [188] metastases; ^n^ includes primary (*n* = 24) [212] and metastatic (*n* = 30) [210] bladder adenocarcinoma; ^o^ includes sarcomatoid urothelial carcinoma of upper urinary tract (*n* = 17) [216] and urothelial carcinoma of the renal pelvis (*n* = 205) [200,201,207,215]; ^p^ includes pure micropapillary urothelial carcinoma (*n* = 18) [217] and urothelial carcinoma with focal micropapillary features (*n* = 15) [217]; ^q^ includes SNS cases, low-grade (*n* = 28) [218] and high-grade urothelial carcinoma (*n* = 58) [218], and urothelial carcinoma with squamous differentiation (*n* = 10) [154]; * these designations are not in line with the current World Health Organization (WHO) classification; ** this designation is not in line with the current WHO classification. It may exist an overlap between the study populations of Heidenreich et al. [221], Nagore et al. [175] and Nagore et al. [186] and the study populations of Egberts et al. [182] and Egberts et al. [184]; it does not seem to exist an overlap of the study populations of Griewank et al. [91] and Griewank et al. [92]; there is a partial overlap of the study populations of Vinagre et al. [51] and Pópulo et al. [171]; *** this designation is not in line with the current WHO classification. It may exist an overlap between the study populations of Rachakonda et al. [204] and Hosen et al. [222].

**Table 2:** Prevalence of *TERT* and *TERC* amplifications in human tumours. Only the tumour histotypes associated with a frequency of *TERT* and *TERC* amplifications ≥5% will appear in the following table together with its respective number, percentage, and range of mutated cases. Whenever the tumour histotypes were associated with a low rate of *TERT* and *TERC* amplifications (<5%) only the total of patients will be shown (complete data available in Supplementary Table S1); the percentages of amplified cases here presented are the same reported by its respective authors, therefore the readers should note that the applied cut-off of copy number alterations may vary among references.

*TERT* Amplifications		
Tumour Type	Amplified Cases (Total)	Amplified Cases in % (Range)
**Tumours of the breast**		
Breast carcinoma, NOS ^a^ [52]	5 (19)	26.3
<5%: phyllodes tumour (*n* = 73) [223].		
**Tumours of the central nervous system**		
Pineoblastoma [224]	1 (1)	100
Classic medulloblastoma [224]	5 (13)	38.5
Nodular medulloblastoma [224]	1 (10)	10.0
Anaplastic medulloblastoma [224]	2 (5)	40.0
Medulloepithelyoma [224]	2 (2)	100.0
Medullomyoepithelyoma [224]	1 (2)	50.0
Ewing sarcoma/peripheral primitive neuroectodermal tumour [224]	4 (8)	50.0
<5%: medullomyoblastoma (*n* = 1) [224].		
**Tumours of the digestive system**		
Colorectal carcinoma, NOS [225]	31 (64)	48.4
Hepatocellular carcinoma, NOS ^b^ [99,103,226]	27 (181)	14.9 (3.4–72.2)
**Tumours of the endocrine system**		
Adrenal cortical carcinoma [45]	11 (75)	14.7
**Tumours of the female reproductive organs**		
Ovarian serous cystadenocarcinoma [45]	6 (27)	22.2
Cervical intraepithelial neoplasia (CIN) 2 [227]	6 (10)	60
Cervical intraepithelial neoplasia (CIN) 3 [227]	7 (8)	87.5
Cervical carcinoma, NOS **^c^** [52,227]	7 (14)	50.0
<5%: cervical intraepithelial neoplasia (CIN) 1 (*n* = 5) [227].		
**Tumours of the head and neck**		
Pharyngeal/laryngeal squamous cell carcinoma, NOS [228]	8 (81)	9.9
<5%: oral squamous cell carcinoma (*n* = 191) [228].		
**Tumours of the lung**		
Lung carcinoma, NOS ^d^ [52]	8 (21)	38.1
Lung adenocarcinoma, NOS [45,229,230]	97 (529)	18.3 (13.2–75.0)
Lung squamous cell carcinoma, NOS [45,229,230]	59 (240)	24.6 (13.8–63.9)
Lung large cell carcinoma [230]	2 (5)	40.0
Mixed histology lung tumours [230]	1 (5)	20.0
**Tumours of the peripheral nervous system**		
Neuroblastoma, NOS [52]	1 (8)	12.5
**Tumours of the skin**		
Acral-lentiginous melanoma [231,232]	14 (60)	23.3 (20.1–29.4)
Melanoma ^e^ [233]	4 (10)	40.0
Merkel cell carcinoma [192]	11 (14)	78.6
<5%: desmoplastic melanoma (*n* = 62) [181].		
**Tumours of the urinary system**		
Urothelial invasive bladder carcinoma [234]	2 (3)	66.7
<5%: urothelial non-invasive bladder cancer (*n* = 15) [234].		
***TERC*** **amplifications**		
**Tumours of the digestive system**		
Oesophageal carcinoma, NOS [45]	36 (168)	21.4
**Tumours of the female reproductive organs**		
Ovarian carcinoma ^f^ [45,235] Cervical intraepithelial neoplasia (CIN) 1 [227,236,237] Cervical intraepithelial neoplasia (CIN) 2 [227,236,237] Cervical intraepithelial neoplasia (CIN) 3 [227,236,237] Cervical carcinoma ^g^ [227,236,237,238,239,240]	13 (35) 10 (41) 29 (44) 51 (58) 53 (90)	37.1 (22.2–87.5) 24.4 (21.1–40.0) 69.0 (50.0–90.0) 87.9 (81.5–100.0) 58.9 (6.1–100.00)
**Tumours of head and neck**		
Absence of histotypes with a frequency of *TERC* amplifications equal or higher than 5%.		
<5%: squamous cell carcinoma of the head and neck, site not-specified (*n* = 31) [238].		
**Tumours of the lung**		
Lung carcinoma, NOS [238] Lung squamous cell carcinoma, NOS [45]	1 (9) 68 (167)	11.1 40.7

^a^ Includes poorly differentiated (*n* = 11) [52] and moderately differentiated (*n* = 8) [52] carcinomas; ^b^ includes moderately to highly differentiated hepatocellular carcinoma (*n* = 34) [226] and poorly differentiated hepatocellular carcinoma (*n* = 12) [226]; ^c^ includes invasive squamous carcinoma (*n* = 9) and in-situ adenocarcinoma (*n* = 1), of which one is well-differentiated, six are moderately differentiated and two are poorly differentiated [52]; ^d^ includes non-small cell lung cancer (*n* = 13) [52] and small cell lung cancer (*n* = 11) [52]; only 21 were analysed; ^e^ includes conventional (*n* = 7), fatal spitzoid (*n* = 1) and melanoma arising in giant congenital naevi (*n* = 2); ^f^ includes serous cystadenocarcinoma (*n* = 27) [45] and carcinoma, NOS (*n* = 8) [235]; ^g^ includes squamous cell carcinoma (*n* = 20) [236,237], adenocarcinoma, NOS (*n* = 12) [240], mucinous adenocarcinoma (*n* = 2) [236], minimal deviation adenocarcinoma (*n* = 1) [236], squamous cell carcinoma and adenocarcinoma (*n* = 4) [227], and carcinoma, NOS (*n* = 33) [238]. It does not seem to exist an overlap of the study populations of Andersson et al. (2006) [240] and Andersson et al. (2009) [237]. The designation ‘NOS’ (not otherwise specified) was applied to the tumours in which a specific histotype was not available.

**Table 3:** Association of the most common *TERT* and *TERTp* polymorphisms with the risk of developing cancer.

***TERT*** **Polymorphism**	**Cancers with Higher Risk of Development**
rs2736100	Acute lymphoblastic leukaemia (paediatric); myeloproliferative neoplasms; bladder, cervical, colorectal, gastric, lung and pancreas (exocrine) cancers, NOS; gliomas; oral squamous cell and papillary thyroid carcinomas [241,242,243,244,245,246,247,248,249,250,251,252,253,254,255,256,257,258,259,260,261,262,263,264,265,266,267,268,269,270]
rs2736098	Bladder, breast, cervical, colorectal, lung, pancreas (exocrine) and prostate cancers, NOS; basal cell, hepatocellular and nasopharyngeal carcinomas, NOS [252,270,271,272,273,274,275,276,277]
rs2853676	Breast, gastric, lung, prostate and ovary cancers, NOS; gliomas, NOS; melanomas, NOS [243,247,278,279,280,281,282,283,284,285,286,287]
**TERT Promoter Polymorphism**	**Cancer Types with Higher Risk of Development**
rs2853669	Bladder, breast, gastric, lung and prostate cancers, NOS; gliomas, NOS; hepatocellular carcinomas, NOS; melanomas, NOS [68,69,76,80,162,188,222,265,275,288,289,290,291,292,293]
rs2735940	Acute lymphoblastic leukaemia (paediatric); gastric and lung cancers, NOS [256,265,294]

The designation ‘NOS’ (not otherwise specified) were applied to the tumours in which a specific histotype was not available.

**Table 4:** Prevalence of alternative lengthening of telomeres (ALT) in human tumours. Only the tumour histotypes associated with ALT-positive phenotype in a frequency ≥5% will appear in the following table together with its respective number, percentage, and range of mutated cases. Whenever the tumour histotypes were associated with a low rate of ALT-positive phenotype (<5%) only the total of patients will be shown (complete data available in Supplementary Table S1).

Tumour Type	Positive Cases (Total)	Positive Cases in % (Range)
**Tumours of the breast**		
Absence of histotypes with a frequency of ALT equal or higher than 5%.		
<5%: invasive lobular carcinoma (*n* = 27) [55], tubular carcinoma (*n* = 9) [55], carcinoma with medullary features (*n* = 55) [55], mucinous carcinoma (*n* = 15) [55] and ductal carcinoma (*n* = 271) [55].		
**Tumours of the central nervous system**		
Diffuse astrocytoma, NOS [55,295,296]	22 (42)	52.4 (27.3–63.0)
Anaplastic astrocytoma, NOS [55,295,296,297,298]	63 (143)	53.8 (26.4–100.0)
Astrocytoma, NOS [299]	17 (50)	34.0
Anaplastic paediatric astrocytoma, NOS [296]	26 (88)	29.5
Paediatric glioblastoma, NOS [55,300]	17 (57)	29.8 (12.0–43.8)
Glioblastoma, NOS ^a^ [55,295,297,298,301,302,303]	147 (953)	15.4 (11.4–50.0)
Oligodendroglioma, NOS [55]	8 (40)	20.0
Anaplastic pleomorphic xanthoastrocytoma [295]	2 (2)	100.0
Choroid plexus carcinoma **^b^** [300]	7 (31)	22.6
Ewing sarcoma/peripheral primitive neuroectodermal tumour ^b^ [300]	5 (43)	11.6
<5%: paediatric anaplastic astrocytoma (*n* = 24) [296], pilocytic astrocytoma (*n* = 45) [295,300], pleomorphic xanthoastrocytoma (*n* = 8) [300], ependymoma (*n* = 95) [300], choroid plexus papilloma (*n* = 24) [300], ganglioglioma (*n* = 8) [300], medulloblastoma, NOS [55,300], atypical teratoid/rhabdoid tumour (*n* = 38) [300], schwannoma (*n* = 44) [55], meningioma (*n* = 46) [55].		
**Tumours of the digestive system**		
Hepatocellular carcinoma, NOS [55]	8 (121)	6.6
Chromophobe hepatocellular carcinoma with abrupt anaplasia [304]	11 (12)	91.7
Gastric adenocarcinoma, NOS [55,305]	16 (197)	8.1 (0.0–38.1)
<5%: oesophageal adenocarcinoma (*n* = 106) [55], oesophageal small cell neuroendocrine carcinoma (*n* = 1) [55], oesophageal squamous cell carcinoma (*n* = 29) [55], adenocarcinoma of the small intestine, NOS (*n* = 215) [55], colon adenoma, NOS (*n* = 136) [55], colon adenocarcinoma, NOS (*n* = 126) [55], hepatocellular adenoma, NOS (*n* = 17) [55], extrahepatic cholangiocarcinoma (*n* = 33) [55], adenocarcinoma of the gallbladder, NOS (*n* = 60) [55], pancreatic ductal adenocarcinoma (*n* = 448) [55], gastrointestinal carcinoid tumours, NOS/SNS (*n* = 47) [306] and carcinoid tumour, NOS/SNS (*n* = 32) [55].		
**Tumours of the endocrine organs**		
Medullary thyroid carcinoma [307]	11 (42)	26.2
Adrenal cortical carcinoma [308]	3 (24)	12.5
Extra-adrenal paranganglioma [55]	1 (8)	12.5
Pancreatic neuroendocrine tumour, NOS [306,309,310,311,312,313,314]	255 (849)	30.0 (14.9–61.0)
<5%: thyroid adenoma (*n* = 34) [55], papillary thyroid carcinoma, NOS (*n* = 47) [55,299], follicular thyroid carcinoma (*n* = 52) [55], parathyroid adenoma (*n* = 38) [55], adrenal adenoma (*n* = 14) [55], and phaeochromocytoma (*n* = 67) [55].		
**Tumours of the female reproductive system**		
Diffuse malignant peritoneal mesothelioma ^c^ [315]	10 (38)	26.3
Uterine carcinosarcoma [316]	8 (16)	50.0
Uterine leiomyosarcoma [316]	7 (8)	87.5
Uterine serous carcinoma [55]	3 (41)	7.3
Uterine stromal sarcoma [316]	4 (17)	23.5
<5%: ovarian serous carcinoma, NOS (*n* = 205) [55], ovarian mucinous carcinoma (*n* = 21) [55], ovarian endometrioid carcinoma (*n* = 72) [55], ovarian clear cell carcinoma (*n* = 56) [55], uterine endometrioid carcinoma (*n* = 64) [55], uterine clear cell carcinoma (*n* = 3) [55], uterine mixed mesodermal tumour (*n* = 4) [55], cervical squamous cell carcinoma (*n* = 152) [55] and cervical adenocarcinoma, NOS (*n* = 19) [55].		
**Tumours of the head and neck**		
Absence of histotypes with a frequency of ALT equal or higher than 5%.		
<5%: laryngeal squamous cell carcinoma (*n* = 29) [55], oral squamous cell carcinoma, NOS (*n* = 41) [55], salivary gland cylindroma (*n* = 28) [55], salivary gland carcinoma, NOS (*n* = 98) [55], pleomorphic adenoma (*n* = 45) [55] and Warthin tumour (*n* = 23) [55].		
**Tumours of haematopoietic and lymphoid tissues**		
Absence of histotypes with a frequency of ALT equal or higher than 5%.		
<5%: diffuse large B-cell lymphoma (DLBCL), NOS (*n* = 10) [55], nodular sclerosis classic Hodgkin lymphoma (*n* = 23) [55], mixed-cellularity classic Hodgkin lymphoma (*n* = 17) [55] and other subtypes of non-Hodgkin lymphoma, NOS (*n* = 54) [55].		
**Tumours of the lung, pleura, thymus and heart**		
Absence of histotypes with a frequency of ALT equal or higher than 5%.		
<5%: lung tumours (adenocarcinoma, NOS (*n* = 153) [55], papillary carcinoma (*n* = 15) [55], bronchoalveolar carcinoma, NOS (*n* = 40) [55], squamous cell carcinoma (*n* = 100) [55], small cell neuroendocrine carcinoma (*n* = 63) [55], carcinoid tumour, NOS (*n* = 3) [55], large cell carcinoma (*n* = 35) [55], other hystotypes, NOS (*n* = 15) [55] and thymoma, NOS (*n* = 37) [55].		
**Tumours of the peripheral nervous system**		
Neuroblastoma, NOS [55,317]	62 (124)	50.0 (9.1–58.8)
<5%: ganglioneuroma (*n* = 3) [55].		
**Tumours of the skin**		
Malignant melanoma, NOS [55]	7 (106)	6.6
<5%: basal cell carcinoma (*n* = 57) [55], squamous cell carcinoma (*n* = 56) [55], benign naevus, NOS (*n* = 12) [55] and benign adnexal tumour (*n* = 15) [55].		
**Tumours of the soft tissues**		
Liposarcoma, NOS [55,299,318]	21 (84)	25.0 (23.5–33.3)
Dedifferentiated liposarcoma [319,320,321]	35 (106)	33.0 (26.9–47.6)
Myxoid liposarcoma ^d^ [319,320,321]	20 (107)	18.5 (5.0–30.0)
Pleomorphic liposarcoma [320]	21 (26)	80.8 (72.7–100)
Fibrosarcoma and variants [55,299]	5 (23)	21.7 (14.3–100.0)
Myxofibrosarcoma [322]	19 (25)	76.0
Leiomyosarcoma [55,299,323]	91 (161)	56.5 (33.3–61.5)
Rhabdomyosarcoma, NOS [55,299]	2 (39)	5.1 (0.0–5.7)
Embryonal rhabdomyosarcoma [322,324]	7 (24)	29.2 (12.5–37.5)
Epithelioid haemangioendothelioma [325]	1 (7)	14.3
Angiosarcoma [55,325]	18 (79)	22.8 (11.1–24.3)
Neurofibroma [55]	2 (2)	100.0
Malignant peripheral nerve sheath tumours [55,320,326]	47 (75)	62.7 (0.0–79.2)
Epithelioid sarcoma [55,299,322]	2 (14)	14.3 (0.0–33.3)
Alveolar soft part sarcoma [299,322]	1 (8)	12.5 (0.0–25.0)
Chondrosarcoma, NOS [55,299]	1 (7)	14.3 (0.0–33.3)
Osteosarcoma ^e^ [299,327,328]	109 (173)	63.0 (46.6–79.6)
Malignant fibrous histiocytic tumour * [299,329]	39 (75)	52.0 (32.6–80.0)
Undifferentiated pleomorphic sarcoma ^f^ [55,322]	55 (86)	64.0 (63.5–64.7)
Radiation-associated sarcoma, NOS [322]	3 (15)	20.0
<5%: lipoma (*n* = 1) [55], solitary fibrous tumours (*n* = 7), dermatofibrosarcoma protuberans (*n* = 9) [322], benign fibrous hystiocytoma (*n* = 16) [55], giant cell tumour of the tendon sheath (*n* = 22) [55], alveolar rhabdomyosarcoma (*n* = 23) [322,324], capillary haemangioma (*n* = 32) [55], Kaposi’s sarcoma (*n* = 55) [55], gastrointestinal stromal tumours (*n* = 50) [55,322], Ewing sarcoma (*n* = 63) [55,322,327], synovial sarcoma (*n* = 24) [299,322], clear cell sarcoma (*n* = 5) [55], extraskeletal myxoid chondrosarcoma (*n* = 2) [322].		
**Tumours of the urinary system and male genital system**		
Chromophobe renal cell carcinoma [55]	4 (47)	8.5
Nephroblastoma [330]	26 (32)	81.3
Sarcomatoid renal carcinoma [55]	2 (27)	7.4
Bladder small cell neuroendocrine carcinoma [55]	3 (13)	23.1
Non-seminomatous germ cell tumour, NOS [55]	7 (46)	15.2
<5%: clear cell renal cell carcinoma (*n* = 117) [55], papillary renal cell carcinoma (*n* = 86) [55], renal oncocytoma (*n* = 18) [55], urothelial muscle invasive (*n* = 150) [55] and non-muscle invasive bladder carcinoma (*n* = 38) [55], non-invasive papillary urothelial carcinoma (*n* = 5) [55], sarcomatoid bladder carcinoma (*n* = 1) [55], squamous cell carcinoma of the bladder (*n* = 2) [55], prostate adenocarcinoma (*n* = 1152) [55], prostate small cell neuroendocrine carcinoma (*n* = 24) [55] and seminoma (*n* = 48) [55].		

^a^ Does not include paediatric glioblastoma; ^b^ includes paediatric patients; ^c^ includes epithelial tubulopapillary (*n* = 5) [315], epithelial solid (*n* = 30) [315] and biphasic (mixed epithelial and sarcomatoid) (*n* = 3) [315]; ^d^ includes round-cell myxoid liposarcoma (*n* = 45) [319,320]; ^e^ includes osteoblastic (*n* = 8) [55], chondroblastic (*n* = 3) [55] and periosteal (*n* = 1) [55] osteosarcoma, and osteosarcoma, NOS (*n* = 32) [55]; ^f^ includes cases classified as malignant fibrous histiocytic tumour [55]; * this designation is not in line with the current WHO classification. The designation ‘NOS’ (not otherwise specified) and ‘SNS’ (site not specified) were applied to the tumours in which a specific histotype was not available.

**Table 5:** Molecular associations, prognostic and clinical implications of telomere maintenance mechanisms in human tumours.

**Hepatocellular Carcinomas**
*TERTp* mutations: marker of malignant progression [95,331,332].
**Well-differentiated thyroid carcinomas**
*TERTp* mutations: association with larger tumours, older patient age, higher tumour stage, tumour recurrence, and distant metastases [51,116,119,126,333]; association with *BRAFV600E* mutations [117,126,141,146].
**Pancreatic neuroendocrine tumours**
*TERTp* mutations: association with hereditary syndromes [57].
ALT: association with protein loss and mutation of ATRX/DAXX [311,312,314]; indicator of more aggressive disease, metastases and worse patient survival [309,311,312,334].
**Diffuse astrocytic and oligodendroglial tumours**
*TERTp* mutations: correlation with older patient age, higher tumour grade, tumour progression and worse overall survival [62,65,80,98]; combination of *TERTp* and *IDH* mutations for survival assessment: *TERTp* and *IDH* concomitant mutations confer the longest overall patient survival; *TERTp* mutations alone confer the lowest [61,71,73,74,78,79].
*TERT* and *TERTp* polymorphisms: rs2736100 [335] and rs2853676 [336] affect the risk of tumour development; association of *TERTp* rs2853669 status with worse prognosis and worse survival in GB patients [68,76,80]; modifying effect of rs2853669 on *TERTp* mutations [65,68,76,80].
ALT: association with ATRX loss [295,296,298,337]; identification of less aggressive GBs with longer patient survival [298,301,303]; mutual exclusivity with *TERTp* mutations [49,50].
Epigenetic mechanisms: methylation of *TERT* promoter as a potential biomarker for malignancy in paediatric gliomas [338].
**Neuroblastomas**
*TERT* rearrangements: indicator of poor prognosis, particularly in combination with *MYCN* amplification [54,339,340,341].
ALT: association with chemoresistant tumours with unfavourable prognosis [342].
**Uterine cervical lesions**
*TERT* and *TERC* amplifications: early identification of patients with low-grade lesions and higher progression risk in routinely liquid based cytology by Pap smears [227,236,237].
**Cutaneous melanomas**
*TERTp* mutations: association with male gender, older patient age, tumour ulceration, higher Breslow’s thickness, and worse overall survival [92,175,179,182,184,221]; association with *BRAFV600E* mutations; combination used to identify tumours with aggressive behaviour [175,179,186].
*TERT* and *TERTp* polymorphisms: association of *TERTp* polymorphism rs2853669 with *TERTp* mutations identify patients at risk of aggressive disease [175,188,293].
**Liposarcomas**
ALT: association with ATRX loss, disease progression and poor clinical outcome [319,320].
**Urothelial bladder carcinomas**
*TERTp* mutations: association with increased disease recurrence and reduced patient survival [209,215,222]; useful biomarker for patient screening as a non-invasive diagnostic and follow-up tool [206,209]; combination with *FGFR3* mutations to identify tumours with poor prognosis [222].
*TERT* amplification: potential biomarker to identify high-risk patients with disease progression [234].
*TERT* and *TERTp* polymorphisms: association of *TERTp* rs2853669 with tumour recurrence and worse patient survival [204].

CIN: cervical intraepithelial neoplasia; GB: glioblastoma; ALT: alternative lengthening of telomeres; TERC: telomerase RNA component; TERT: telomerase reverse transcriptase.

**Table 6:** Distribution of absent/low frequency telomere maintenance mechanisms (TMMs) in prevalent human tumours.

	*TERT* Promoter Mutations, % (Total)	*TERT* and *TERC* Amplifications % (Total)	ALT, % (Total)
**Tumours of the breast**			
	0 (210)	26 (19)	0–3.7 (377)
**Tumours of the digestive system**			
Stomach	0 (543)	N.A.	0–8 (197)
Small intestine	N.A.	N.A.	0 (215)
Colon and rectum	0 (22)	48 (64)	0 (126)
Exocrine pancreas	0 (95)	N.A.	0 (448)
**Tumours of haematopoietic and lymphoid tissues**			
	2 (424)	N.A.	0 (104)
**Tumours of the lung**			
	1 (611)	9 (976)	0 (424)
**Tumours of the male genital organs**			
Prostate	0 (99)	N.A.	0 (1176)

N.A.: not available. Tumour histotypes are depicted in Table 1, Table 2 and Table 4. ALT: alternative lengthening of telomeres; TERC: telomerase RNA component; TERT: telomerase reverse transcriptase.

## Supplementary Materials

The following are available online at http://www.mdpi.com/2073-4425/9/5/241/s1, Table S1: Prevalence of different TMMs by organ/system (complete data).
